# Ultrasound-assisted brain delivery of nanomedicines for brain tumor therapy: advance and prospect

**DOI:** 10.1186/s12951-022-01464-z

**Published:** 2022-06-16

**Authors:** Shuo Zhang, Shuai Zhang, Siyuan Luo, Peng Tang, Mingxi Wan, Daocheng Wu, Wei Gao

**Affiliations:** 1 The Key Laboratory of Biomedical Information Engineering of Ministry of Education, School of Life Science and Technology, Xi’an Jiaotong University, Xi’an, 710049 People’s Republic of China; 2 Department of Anesthesiology and Center for Brain Science and Center for Translational Medicine, The First Affiliated Hospital of Xi’an Jiaotong University, Xi’an, People’s Republic of China

**Keywords:** Ultrasound, Blood–brain barrier, Nanomedicines, Brain Tumor

## Abstract

Nowadays, brain tumors are challenging problems, and the key of therapy is ensuring therapeutic drugs cross the blood–brain barrier (BBB) effectively. Although the efficiency of drug transport across the BBB can be increased by innovating and modifying nanomedicines, they exert insufficient therapeutic effects on brain tumors due to the complex environment of the brain. It is worth noting that ultrasound combined with the cavitation effect of microbubbles can assist BBB opening and enhance brain delivery of nanomedicines. This ultrasound-assisted brain delivery (UABD) technology with related nanomedicines (UABD nanomedicines) can safely open the BBB, facilitate the entry of drugs into the brain, and enhance the therapeutic effect on brain tumors. UABD nanomedicines, as the main component of UABD technology, have great potential in clinical application and have been an important area of interest in the field of brain tumor therapy. However, research on UABD nanomedicines is still in its early stages despite the fact that they have been associated with many disciplines, including material science, brain science, ultrasound, biology, and medicine. Some aspects of UABD theory and technology remain unclear, especially the mechanisms of BBB opening, relationship between materials of nanomedicines and UABD technology, cavitation and UABD nanomedicines design theories. This review introduces the research status of UABD nanomedicines, investigates their properties and applications of brain tumor therapy, discusses the advantages and drawbacks of UABD nanomedicines for the treatment of brain tumors, and offers their prospects. We hope to encourage researchers from various fields to participate in this area and collaborate on developing UABD nanomedicines into powerful tools for brain tumor therapy.

**Graphical Abstract:**

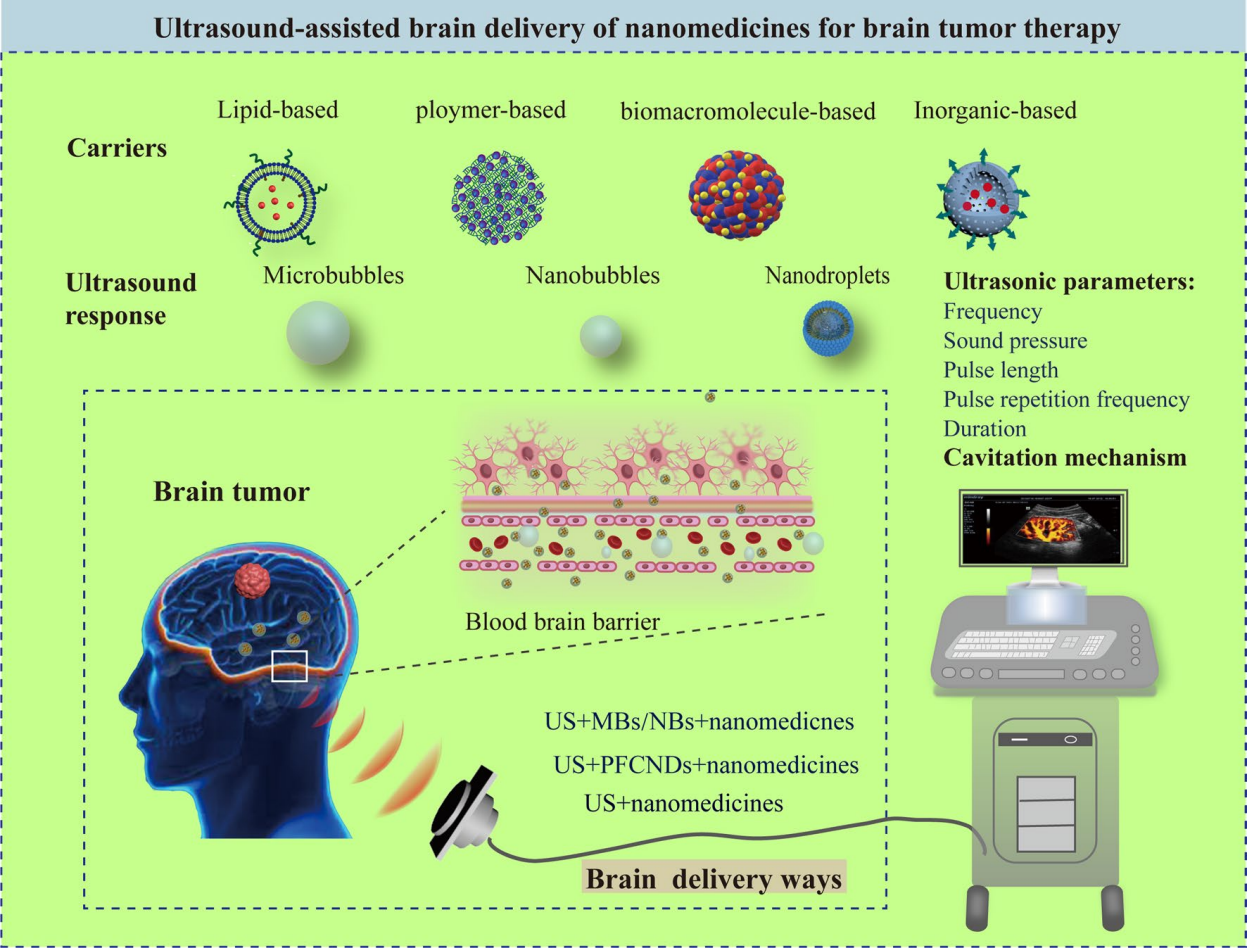

## Introduction

Brain tumors are challenging problems cause a number of health risks. The critical reason is the limited therapeutic effect due to the difficulty in performing surgical resection, low drug effectiveness, and high tumor recurrence rate [1]. Moreover, most therapeutic strategies are still based on therapeutic drugs, including traditional small molecule drugs, such as cisplatin and temozolomide [2, 3] and biological macromolecular drugs, such as nucleic acids, peptides, proteins, and monoclonal antibodies [4]. The blood–brain barrier (BBB) in the brain prevents 98% of small molecular drugs and entire macromolecular drugs from entering the brain, which is made up of three types of cells in the cerebral microvascular system: endothelial cells, astrocytes, and pericytes [5]. Astrocyte end feet adhere to the basement membrane that surrounds endothelial cells and pericytes, which is essential for the induction and maintenance of the BBB tight junctions [6]. Furthermore, tight junctions between endothelial cells constitute a cell barrier that can selectively obstruct the entry of most substances from the bloodstream into the brain. Hence, the key for effective brain tumor therapy is enabling drugs to pass through the BBB.

Developments in nanotechnology have led to renewed interest in nanomedicines, which are nanoscale drugs for diagnosing, monitoring, controlling, preventing, and treating diseases. They boost the efficiency of drugs in the brain relative to that of free drugs, but the presence of the BBB remains a major challenge in brain drug delivery [7]. The efficiency of drug transport across the BBB can be increased by innovating and modifying nanomedicines. For instance, targeting molecules that can be recognized by a receptor on the BBB’s surface have been conjugated with nanomedicines to facilitate the delivery of drugs across the BBB [8, 9]. Cationic proteins or cell-penetrating peptides are used to mediate cell endocytosis and enable drug transport across the BBB [10, 11]. Moreover, nanoviral vectors can facilitate drug delivery to the brain to some extent [12, 13]. Although these approaches have improved the efficiency of drug delivery to the brain, they do have major limitations: (1) BBB components have many receptors, which must be paired with specific targeting molecules. Thus, combining a variety of targeting molecules on nanomedicines complicates nanomedicines manufacture; (2) Owing to restricted drug transport through the BBB, an immediate therapeutic is difficult to observe; (3) The biochemical delivery method may cause irreversible damage to the BBB. Therefore, these nanomedicines exert insufficient therapeutic effects on brain tumors and cannot be used in clinics.

Ultrasound is an established biomedical technology routinely employed for disease detection and treatment. In 1950, Bakay et al. was the first to report that high-intensity focused ultrasound (HIFU) can induce BBB damage [14]. In 2003, Vykhodtseva et al. discovered that ultrasound combined with microbubbles (USCM) can safely and effectively open the BBB, thereby establishing a new field of transcranial ultrasound treatment for brain diseases [15]. Since then, a number of studies have demonstrated that ultrasound assists BBB opening and enhances brain delivery of nanomedicines. We called this ultrasound assisted drug brain delivery technology (abbreviated as UABD technology) and associated nanomedicines are regard as UABD nanomedicines. This technology includes the following ways: only using ultrasound to open BBB to assist drugs or nanomedicines into the brain as well as using ultrasound combined with cavitation effect of microbubbles to open BBB to enable drugs or nanomedicines into the brain. Cavitation effect is that the bubbles or substances produce bubbles in the liquid when the ultrasonic intensity is sufficient and the bubbles increase continuously with the expansion cycle of ultrasonic wave until they reach the unstable size, and then collapse violently, resulting in a variety of biological and thermal effects [16]. Generally, the bubbles that combine ultrasound to produce cavitation effect include microbubbles (MBs), nanobubbles (NBs) and perfluorocarbon nanodroplets (PFCNDs) [17, 18]. Among them, opening BBB directly by ultrasound will cause irreversible brain damage, which has not been considered for clinical use. Similarly, although the direct use of ultrasound to open the BBB to make nanomedicines enter the brain has been investigated, its therapeutic effect is limited to a certain extent for special diseases. Herein the UABD nanomedicines mainly referred to those nanomedicines that are used for entering the brain by ultrasound combined with MBs to open BBB. It is found that ultrasound has significant advantages over microwave [19, 20], laser [21, 22], and electromagnetic [23, 24] field for brain diseases’ therapy. The following are some of the advantages of UABD technology:

1. Brain delivery efficiency of drugs is greatly improved because of BBB opening. Ultrasound safely and briefly opens the BBB, fundamentally improving the efficacy of nanomedicines transport to the brain via the BBB. USCM opens the BBB through the cavitation effect by ultrasound and MBs [25]. Once the BBB is opened, nanomedicines accumulating on its side rapidly passed through, allowing treatments to act in the affected brain region.
2. Decreased trauma and increased security in this process. Ultrasound is a widely used method in clinical practice because it causes negligible or no damage to normal tissues during disease diagnosis and therapy [26]. Numerous studies have demonstrated that the USCM technology can open the BBB in specific brain areas noninvasively, reversibly, and repeatedly and the BBB normally recovers within 24 h without causing neuronal injury [27].
3. Drug release can be controlled in the brain. Ultrasound has drug-responsive release qualities. Ultrasound accelerates the release of drugs from liposomes [28, 29], capsules [30], MBs, and PFCNDs [31, 32]. By manipulating the ultrasound’s properties (intensity, time, frequency, and duty cycle), the release of drugs can be efficiently released in time and space, and hence the therapeutic efficacy of the drugs improves.

UABD nanomedicines, as the key components of the UABD technology, have become a major subject of interest in brain tumor therapy, and studies on the treatment of brain tumors and other diseases, such as stroke, Alzheimer's disease (AD), Parkinson's disease, and Huntington's dance syndrome, have shown promising results. UABD nanomedicines have become an extremely challenging and promising research direction in the biomedical field. To date, research on UABD nanomedicines is still in an early stage; its theoretical foundations and associated technologies have not been extensively investigated, and no clinical applications of UABD nanomedicines have been reported, despite the fact that some beneficial results have been obtained with UABD nanomedicines. The primary reasons are as follows: (1) UABD nanomedicines is an interdisciplinary area that involves collaboration among researchers from different disciplines (e.g., materials, medicine, ultrasonography, and brain science), and associated hurdles may be insurmountable; (2) Owing to the complexity of the brain’s environment, designing nanomedicines that are suitable for UABD technology, efficiently penetrate the BBB, and exert therapeutic effects against brain tumors are challenging; (3) Although a huge number of brain science studies have been recorded in the biomedical field. Research on specially designed nanomedicines that are compatible with the UABD technology (UABD nanomedicines) and can deliver drugs to the brain effectively remains limited. Numerous issues about UABD nanomedicines should be identified, such as interaction between nanomedicines materials and UABD and the mechanism by which nanomedicines penetrate the BBB. Hence, a comprehensive review of UABD nanomedicines for brain tumor therapy is urgently needed.

**Fig. 1:**
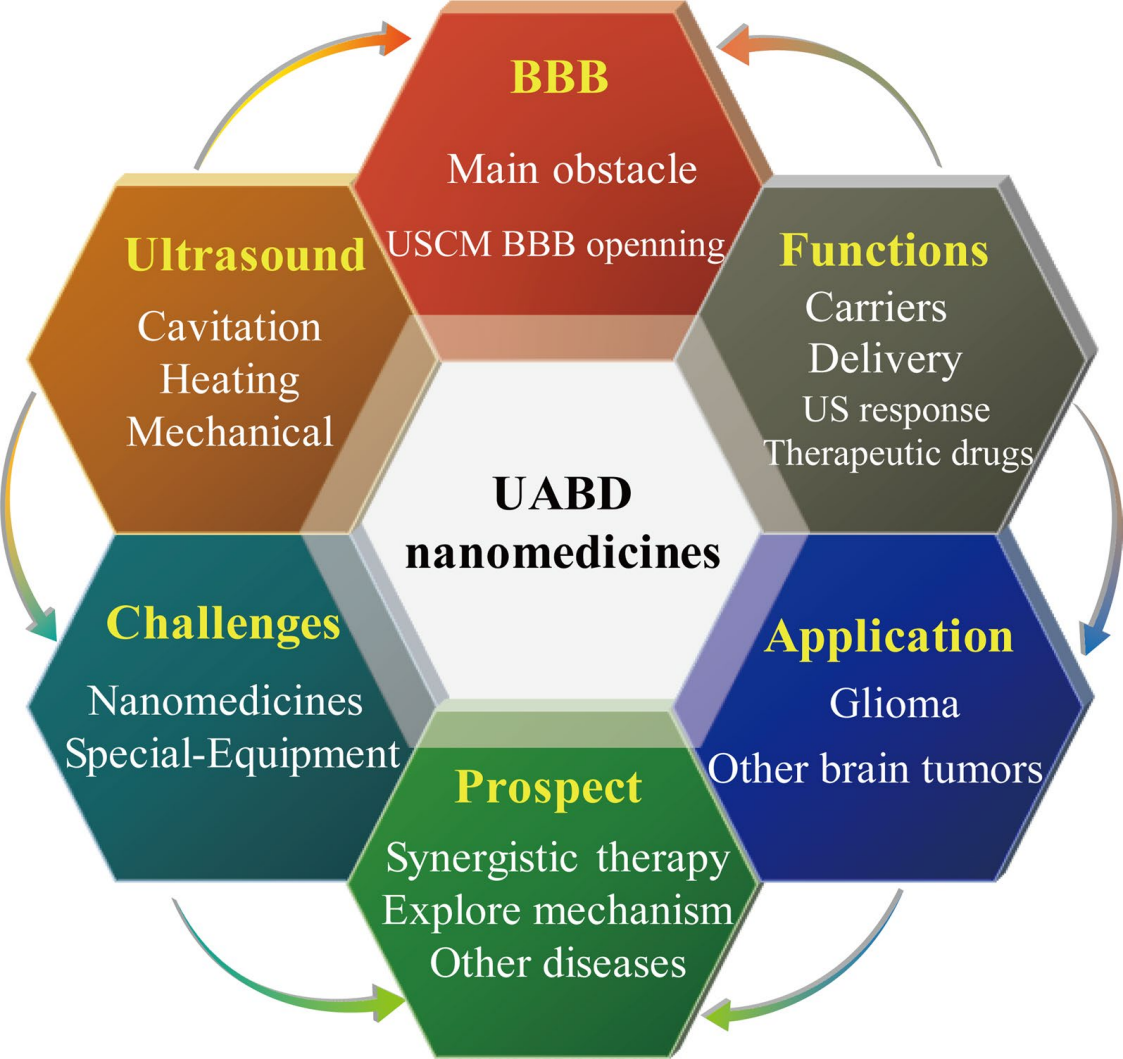
The main contents of this review, including the introduction of UABD nanomedicines and their applications, challenges and prospects

As shown in Fig. 1, this review discusses preliminary research on UABD nanomedicines, intends to introduce the current state of research in this field, summarize the related research progress on BBB opening and controlled release of nanomedicines in the brain; and discuss the properties of special UABD nanomedicines. Then, the uses of UABD nanomedicines in brain tumor treatment are explored; Finally, unresolved problems in this field and prospective research objectives are discussed. The aims are to stimulate collaboration among academics from other fields, address critical scientific problems with UABD nanomedicines, and develop it as a strong tool for brain tumor treatment.

## USCM BBB opening

### Conditions for USCM BBB opening

Ultrasound is a mechanical wave that has a modest depth of radiation, high accuracy, focusable property and controllable energy. It is widely used in the diagnosis and treatment of numerous illnesses, including brain diseases [33–35]. In 2015, Scientists in Sunnybrook Health Science Center of Canada used USCM to temporarily open the BBB of patients with AD and found that harmless dyes can infiltrate the brain. The BBB was completely restored to its original state without neuronal damage within 24 h [27]. Similarly, a recent Phase II clinical trial led by Kaplitt et al. demonstrated that Magnetic Resonance (MR)-guided USCM can open the BBB in the hippocampus and entorhinal cortex of patients with AD safely, noninvasively, transiently, repeatably, and intensively [36]. USCM causes minimal injury to normal tissues or nerves, showing potential as a method for brain drug delivery and brain tumor treatment [37, 38].

The precise control of ultrasound parameters is critical for BBB opening by USCM. The pressure and duration of ultrasound are directly related to the opening degree of the BBB, and the ultrasound intensity increases with the increase of ultrasound pressure. Hynen et al. [39] observed the BBB disruption in rabbits under different ultrasound pressures. MRI results showed that ultrasound pressure of 0.4 MPa could cause the rupture of the BBB in about 50% and 90% at 0.8 MPa as well as 100% at 1.4 MPa. The histological results after 4 h of survival showed that about 70–80% of brain tissue necrosis was induced by ultrasound at the ultrasound pressure level of 2.3 MPa or higher. In addition, Chang et al. [40] compared the opening and damage degree of the BBB when the total ultrasonic duration was 30 s, 60 s, 120 s and 300 s, respectively. When the duration of ultrasound was 30 s and 60 s, the BBB opened and ethidium bromide (EB) extravasation increased from 5.87 to 13.72%, and there was no obvious tissue damage in the two groups. The opening degree of the BBB continued to increase when the ultrasound duration reached 120 s, but it also began to produce tissue damage (EB 23.04%; damage score 1) and further increased at 300 s (EB 25.14%; damage score 2). At the same time, when the ultrasound pressure is 0.5 MPa, the BBB opening effect with the pulse length of 10 ms is significantly better than that the pulse length of 1 or 0.1 ms. Therefore, as shown in Table 1, maintaining the ultrasound pressure below 1 MPa can prevent blood vessel wall rupture and bleeding. The ultrasound center frequency is generally maintained at about 1 MHz and the pulse length is kept below 10 ms. It can also be seen from Table 1 that the most frequently employed form of ultrasound is focused ultrasound (FUS), which has a high focusing effect and high penetrating ability.

**Table 1:** Summary of parameters for USCM BBB opening

Carrier system	Types of ultrasound	Parameters of ultrasound				Mechanism	Duration (min)	Refs
		Frequency (MHz)	Sound pressure (MPa)	Pulse length (ms)	PRF (Hz)			
BCNU bubbles	pFUS	10	0.5–4.5	–	10	Cavitation effect	4	[42]
DOX-SPIO-MBs	pFUS	0.4	0.325	–	1	Cavitation effect	1.5	[43]
PFP nanodroplets	pFUS	1.5	0.45; 0.60	6.7	5	Cavitation effect	5	[44]
PEG-PLGA-PFP nanodroplets	cFUS	1	1.0	–	–	Thermal effect; cavitation effect	3	[45]
Nanobubbles	pFUS	0.75 ± 0.05	< 0.5	10	1	Cavitation effect	0.5	[46]
–	LIFUP	1	0.3	–	1	Cavitation effect	2	[47]
Propofol-loaded nanoemulsions	pFUS	1	0.5	10	0.5	Cavitation effect	2	[48]
–	pFUS	0.69	0.6–0.8	10	1	Cavitation effect	1	[49]
AP-1 Lipo-Dox	HIFUS	1	0.7	–	1	Cavitation effect	–	[50]
CDDP-BPN nanoparticles	cFUS	1.14	0.8	–	–	Cavitation effect	–	[51]
The liposomal (LP)-O.^6^BTG-C_18_ nanoparticles	LIFUP	–	0.28–0.55	10	1	Cavitation effect	3	[52]
BCNU-MBs	pFUS	1	0.5	5	5	Cavitation effect	2	[53]
GDNFp-liposome	pFUS	0.5	0.33	10	1	Cavitation effect	0.5	[54]
MBs	pFUS	0.612	0.55–0.81	10	1	Cavitation effect	1	[55]
Albumin-based nanoclusters	pFUS	1.1	0.30	10	1	Cavitation effect	2	[56]

Abbreviations: *pFUS*, pulsed focused ultrasound; *PRF*, pulse repetition frequency; *LIFUP/HIFUP*, low-intensity/ high-intensity focused ultrasound pulses; *cFUS*, continuous focused ultrasound

We believe that all of the above parameters of ultrasound have the impact on the opening of the BBB. In order to optimize the conditions for ultrasound to open the BBB, we must comprehensively consider various parameters for BBB opening [41]. At present, there are few articles dedicated to the parameters of ultrasound to open the BBB. We considered that mathematical simulation and experiment verification may be a useful method to study the optimization conditions of ultrasonic parameters. Overall, these ultrasonic settings are extremely beneficial for efficient USCM BBB opening. However, the optimization of these critical factors is still being investigated. Currently, instances of particular biological damage induced by ultrasound are few, and safety standards for various parameters remain experimentally unsupported. Hence, researchers may turn their attention to ultrasound parameters and safety regulations in the future.

pFUS, pulsed focused ultrasound; PRF, pulse repetition frequency; LIFUP/HIFUP, low-intensity/ high-intensity focused ultrasound pulses. cFUS, continuous focused ultrasound.

### Cavitation effect (a possible mechanism for USCM BBB opening)

**Fig. 2:**
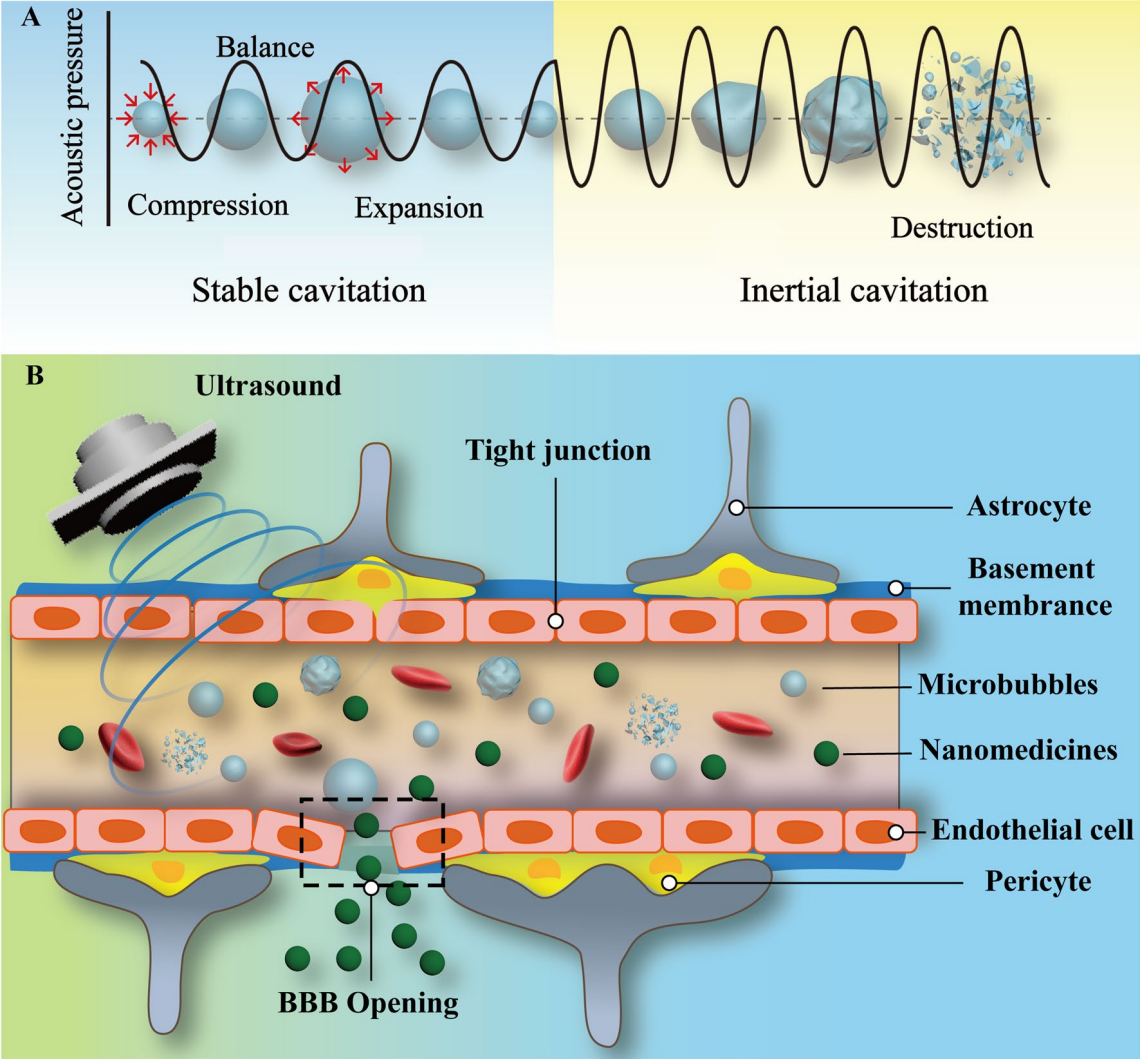
**A** Scheme of cavitation effect. **B** USCM BBB opening

USCM in combination with free or model drugs is an important UABD technology. Although the exact process of USCM BBB opening is unknown, researchers generally believe that the cavitation effect is necessary, as shown in Table 1 [57]. When high-intensity ultrasound is transmitted through a liquid, a void (MBs nucleus) forms, which can oscillate, grow, and collapse rapidly when sound pressure changes [58]. The growth and collapse of MBs is called “acoustic cavitation” [59]. The cavitation effect can be classified into steady-state and inertial cavitation according to the dynamic behavior of MBs exposed to ultrasonic waves. In steady-state cavitation, the radius of a bubble changes periodically in response to ultrasonic frequency and persists for an extended amount of time within the air pressure threshold. Hence, MBs can contract and expand for an extended period when exposed to ultrasound, potentially allowing the opening of the BBB’s tight connection via push–pull mechanisms. Once the ultrasound energy exceeds a particular threshold, MBs are forcefully collapsed and broken, resulting in inertial cavitation (transient cavitation), as shown in Fig. 2 [60]. MBs rupture leads to secondary mechanical processes, such as shock waves generated by liquid ejection and MBs rupture, which further promotes BBB opening [61]. Hence, these biophysical effects are considered crucial driving factors for BBB opening [62, 63]. Yet, the inertial cavitation of MBs induced by FUS exposure can burst vessels or harm nerve tissue. In conclusion, it should minimize inertial cavitation and sustain a longer steady-state cavitation duration during the USCM BBB opening process [64]. Notably, the effects of ultrasound and mechanical effects contribute to the opening of BBB by USCM. These effects occur simultaneously and are difficult to distinguish from one another. For instance, temperature changes can modify the cavitation threshold, and the cavitation effect is usually accompanied by temperature changes [65, 66]. Consequently, the opening of the BBB by USCM may be the result of a combination of several mechanisms [62].

The precise mechanism by which USCM increases the BBB’s permeability has not been clarified because a variety of effects occur concurrently with cavitation, and these effects impedes the identification of numerous physical and chemical changes. As an important UABD technology, many studies have shown that combining USCM with nanomedicines has a good synergistic effect for opening the BBB and delivering drugs to the brain. Nanomedicines may provide cavitation nucleation sites, lowing the threshold of cavitated-assisted brain delivery technology and promoting the formation of cavitation bubbles and thereby enhancing the cavitation effect. Simultaneously, the cavitation effect of ultrasound can facilitate the penetration of nanomedicines into the brain. Thus, cavitation plays a critical role in the relationship between ultrasound and nanomedicines. Meanwhile, the association between nanomedicines and UABD is still being researched, and future study should focus on this area. whereas the relationship between nanomedicines and UABD has not been thoroughly investigated [67].

## UABD nanomedicines

Owing to BBB impediment, the traditional administration dosage is significantly higher than the regular needed dosage for the clinical treatment of brain tumors. A high dosage has a serious adverse effect on brain tissues and greatly limits the usage of a number of drugs [68]. By modifying nanomedicines, UABD technology not only reduces BBB restrictions but also improves drug accumulation in the brain [69]. Generally, the functions of UABD nanomedicines include carriers, delivery, ultrasound response and therapeutic drugs. According to the different types of brain delivery, cavitation materials can be combined with the other two components to form UABD nanomedicines, or they can be used alone to open BBB under the action of ultrasound and deliver nanomedicines to the brain. The ultimate object is to improve the BBB passing efficiency of nanomedicines and enhance the therapeutic effect of brain tumors. We believe that cavitation materials, whether combined with nanomedicines or not, should be as a part of UABD technology. Thus, UABD nanomedicines, as a collection of components based on UABD technology, are vital to the treatment of brain tumors.

### Carriers of the UABD nanomedicines

Owing to the special environment of the brain and the existence of BBB, the carriers of UABD nanomedicines have the higher requirements than that of general nanomedicines. Table 2 shows parameters of the carriers of UABD nanomedicines reported recently. It is found that they mainly include liposomes, polyethylene glycol (PEG) associated polymers and a small amount of inorganic carriers, such as TiO_2_, and nanogolds, etc. The most used carrier of UABD nanomedicines is liposomes [70]. The reason is not only that liposomes has good biocompatibility and biodegradability in vivo, but also they can encapsulate insoluble and soluble drugs as well as fabrication of ultrasonic-sensitive liposomes for ultrasound imaging [71]. Similarly, some natural biomacromolecules with easy degradation and low immunogenicity, such as albumin and DNA, are also used as carriers for UABD nanomedicines. PEG is one of the synthetic polymer compounds that can be used in clinic. It has the characteristics of good biocompatibility, biodegradability in vivo and easy functional modification. As carriers of UABD nanomedicines, it can be endowed with more functions. A small number of inorganic materials such as TiO_2_ and nanogolds have also been reported as the carriers of UABD nanomedicines, but these materials have poor biocompatibility in vivo and are not easy to metabolize in brain, so they are difficult to be used in clinic in the future [72, 73]. This situation shows that the basic requirement of UABD nanomedicines carriers is to quickly pass through the BBB and enter the brain to exert its efficacy under the conditions of biocompatibility and in vivo degradation. Carriers of UABD nanomedicines are selected from the existing drug carriers. Due to the lack of research on the interaction between carriers to BBB and the impact of ultrasonic cavitation on carriers, there is no special carriers designed for UABD nanomedicines at present. We believe that systematic research on the design theory of carriers to adapt to the influence of BBB and ultrasonic cavitation, and the development of new carriers of UABD nanomedicines will become an important research direction in the future.

**Table 2:** The carriers of UABD nanomedicines

Types	Materials	Sizes (nm)	Drugs	Refs
Lipid-based	PEG-PEI	100	RVG-R9& pDNA	[12]
	Hydrogenated soybean L-α-phosphatidylcholine& cholesterol& DSPE-PEG2000;	116.1 ± 30.3	DOX&AP-1	[74]
	DSPE-PEG2000& DPPG& DSPC& PEG4000	50–200	MTX	[75]
	DPPG	180–200	Cisplatin	[76]
	DPPC; DSPE-PEG2000-amine; Chol	105	Gene	[54]
Polymer-based	PEG‑b‑PMNT&PEG‑b‑PCMS	40	RNPs	[77]
	PLGA& DPPC	≤ 60	siRNA	[78]
	SPAnH	~ 10–20	BCNU	[79]
	PEBCA	274	dye	[80]
	PEG-PLA	105.4 ± 33.0; 106.7 ± 33.9; 129.0 ± 41.7	Coumarin 6; DiR; Fe_3_O_4_	[81]
Biomacromolecule-based	Albumin	–	Paclitaxel	[47]
	BPN gene vectors	~ 50	Cy5-labeled plasmid DNA	[82]
	Albumin	100 ~ 200	Ultrasmall iron oxide	[56]
Inorganic-based	AuNPs	~ 10	Cisplatin	[83]
	TiO_2_	210 ± 23	TMZ	[84]

### Delivery of the UABD nanomedicines

Drug brain delivery types of nanomedicines has a significant impact on the therapeutic effect of UABD technology, which has three ways of drug brain delivery: (1) Ultrasound opens the BBB and promotes the entry of nanomedicines into the brain (ultrasound + nanomedicines); (2) Ultrasound combined with MBs/NBs opens the BBB and promotes the entry of nanomedicines into the brain (ultrasound + MBs/NBs + nanomedicines); (3) Ultrasound combined with PFCNDs opens the BBB and promotes the entry of nanomedicines into the brain (ultrasound + PFCNDs+ nanomedicines).

#### Ultrasound + nanomedicines

Nanomedicines are primarily employed in the treatment of solid tumors because they can improve drug permeability and retention (EPR), prolong drug half-life, and improve therapeutic effects [85, 86]. Currently used for treat brain tumors, nanomedicines have a better therapeutic effect for brain tumors than that of free drugs, but they have no satisfactory therapeutic results because of the blockage of the BBB [87]. In 1956, Bakay et al. [14] applied HIFU for the first time to induce BBB opening. Since then, ultrasound’s capability to destroy the BBB has attracted considerable interest. The efficiency of nanomedicines entering the brain can be significantly increased through ultrasound. Eventually, drugs are effectively delivered to the brain’s nidus tissues [11].

For example, Prausnitz et al. [88] injected gadolinium-labeled liposomes loaded with different drugs directly into primate brain tissues and administered them using ultrasonic exposure at 85 kHz and 1200 J/cm^2^. The conductivity of brain tissue increased approximately 10–50 times, and the tissue permeability of medicines increased roughly 25 times relative to those after liposome administration without ultrasound. These findings demonstrated that ultrasonic intervention can greatly facilitate the penetration of nanomedicines into the brain. However, the precise reasons and processes of this occurrence have not been thoroughly investigated. Only a considerable increase in drug delivery rate was observed macroscopically. Furthermore, Rehman et al. [84] loaded temozolomide (TMZ) onto TiO_2_ nanosticks through physical adsorption and established nanomedicines for glioblastoma multiforme (GBM) by using these nanosticks. They revealed that when the BBB is opened by ultrasonic radiation, nanomedicines accumulate and are used more effectively in brain tumors. The ROS generated by the nanomedicines can increase the GBM’s sensitivity to TMZ and diminish tumor cells’ drug resistance to TMZ. According to relevant research, this procedure is frequently accompanied by cerebral hemorrhage, which poses a significant risk of injury to the exceedingly precise brain [89]. Those results showed that using ultrasound in opening the BBB and facilitating the entry of nanomedicines to the brain is not the ideal type for drug brain delivery. Thus, investigations on the direct use of HIFU to induce BBB opening and deliver nanomedicines are rare. This problem of safe BBB opening has not been solved so far and strategy that can prevent HIFU injury is needed.

#### Ultrasound + MBs/NBs + nanomedicines

**Fig. 3:**
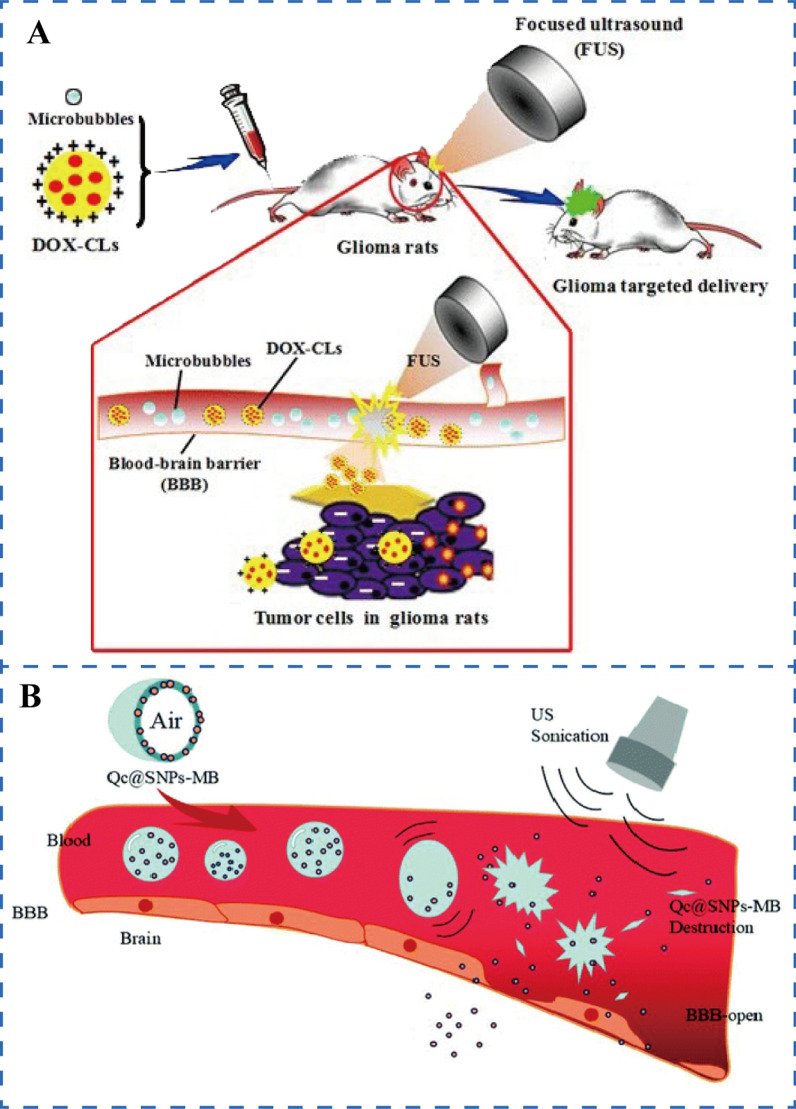
**A** Brain tumor delivery by USCM introduced nanomedicines [124]. **B** The integrated system of MBs and nanomedicines brain delivery assisted by ultrasound [93]

The exclusive use of ultrasound to increase the permeability of nanomedicines to the brain is regularly fraught with danger. MBs were created to somewhat resolve this problem. Ultrasound combined with MBs opens the BBB and facilitates the entry of nanomedicines into the brain is also referred to UABD technology. Vykhodtseva et al. showed that using MBs to open the BBB with UABD technology offers numerous advantages over performing a single HIFU: (1) High level of security. The UABD technology can enable FUS to open the BBB effectively without causing tissue damage; (2) Low energy consumption. The intensity required to induce BBB opening by UABD technology is two orders of magnitude lower than that required by HIFU alone; (3) Rapid recovery. After opening, the BBB can be restored to its previous state in less than 24 h [89, 90]. As a consequence, this type of brain drug administration is more easily applicable to the clinic than the prior methods. Arvanitis et al. [78] used positively charged liposomes as carriers for siRNA-loaded nanomedicines, and transiently modulation of the permeability of the BBB under the cavitation effect produced by FUS combined with MBs improves these nanomedicines delivery in the brain. The study demonstrated that the efficiency of siRNA delivery to the brain tumor microenvironment through this delivery mode is boosted tenfold that in liposomes alone and is considerably higher than the siRNA delivery efficiency reported by Li et al., who used only ultrasound to deliver nanomedicines. Similarly, Davies et al*.* [80] adopted this method to safely induce BBB opening and quantified the relationship between the efficiency of nanomedicine delivery and degree of BBB opening. They discovered that the amount of nanomedicines in the cerebral hemisphere of the UABD treatment group were 7.7 times than that of the control group. This result suggested that nanomedicines delivery is truly dependent on BBB opening. The efficiency of brain delivery is related to the concentration of MBs. In 2007, Treat et al. [91, 120] pioneered the use of this drug delivery type to enhance UABD technology administration by injecting the synthesized liposomal doxorubicin (DOX) nanomedicines and MBs successively. The findings confirm that the concentration of DOX in the brain increased linearly with the dose of MBs exposed to ultrasound. When the MBs were at high doses of (0.2 and 0.5 ml/kg), the DOX concentrations in brain regions treated with ultrasound were 2369 ± 946 and 5366 ± 659 ng/g, whereas the DOX concentrations in the control tissue samples remained at or below 251 ± 119 ng/g. Meanwhile, Lu et al. [124] developed this strategy to inject MBs and DOX-loaded cationic liposome nanomedicines into the tail veins of mice successively, as shown in Fig. 3A. Ultrasound-mediated BBB opening enhances glioma-targeted delivery of DOX, which not only increases the inhibition rate of glioma but also extends the survival period of mice to 81.2 days. Numerous significant advances in the delivery of nanomedicines to the brain through USCM have been made, but vascular or tissue damage has been observed occasionally during UABD technology for brain exposure. For this problem, McDannold et al. [92] designed a closed-loop cavitation control paradigm. The paradigm constructs a dual-transducer system that operates at a low ultrasonic frequency, providing steady cavitation and the suppression of inertial cavitation behavior. The findings of the experiments indicated that the controller can transport a specified number of nanomedicines to the brain without detecting erythrocyte extravasation, laying a foundation for future UABD technology, USCM nanomedicine release control and therapeutic therapy of brain illnesses. However, injecting MBs and nanomedicines separately cause them reach the BBB at inconsistent time. Some researchers combined them by placing nanomedicines on the surface or inside MBs and enabled them to open the BBB while simultaneously releasing nanomedicines during ultrasound exposure. As shown in Fig. 3B, Qin et al. [93] created an integrated system (Qc@SNPs-MB) by embedding quercetin-modified sulfur nanoparticles (Qc@SNPs) in MBs. The results indicated that Qc@SNPs-MB was destroyed immediately after ultrasound exposure, which not only enhanced the permeability of the BBB but also facilitated the transport of nanomedicines, ultimately treating AD effectively.

Similarly, Yeh et al. [132] coupled the vascular endothelial growth factor (VEGF-A) ligand with MBs loaded with 1,3-bis (2-chloroethyl)-1-nitrosourea (BCNU) to prepare an integrated system (VEGF-BCNU-MBs). In vitro tests revealed that this system’s affinity for tumor cells increased 15.98 times (p < 0.001). VEGF-BCNU-MBs not only open the BBB with the assistance of ultrasound but also can improve the distribution of local chemotherapeutic medicines (in the tumor area increased by approximately 50%), extending the survival duration of glioma model rats to 42 days.

Although the UABD technology in combination with nanomedicines has not been tested in clinical trials, UABD technology in combination with free or model drugs have been used in clinics [94]. Thus, UABD for nanomedicines delivery across the BBB has promising clinical applications. However, this method in the brain tumor therapy has two major drawbacks: first, MBs have extremely low half-lives in vivo and circulation time of previous MBs has less than one minute; even now, MBs containing fluorocarbon gases have a circulation time of no more than 10 min. Thus, the ultrasonic cavitation effect and influence on the BBB are decreased, and the degree of medicine accumulation in the brain and the therapeutic effect are subsequently reduced [95, 96]. Second, MBs cannot successfully traverse many biological barriers and may become stuck in the caillary bed of the lungs or other organs because of their huge sizes, and thus tissue injury and subsequent microcirculation obstruction occur [97, 98]. Moreover, the manufacturing of drug-loaded MBs is time consuming and expensive. Developing an alternative material that retains the advantages of MBs and is simple to prepare and affordable is critical.

In view of the issues with MBs, NBs were developed to replace MBs and overcome their adverse effects [99]. “ultrasound + NBs + nanomedicines” can be further divided into two parts: drug-free NBs or drug-loading NBs. Drug-free NBs are used separately from nanomedicines in the process of using UABD technology, while drug-loading NBs and nanomedicines play a therapeutic role as a whole. However, because drug-free NBs have not been reported for brain delivery by ultrasound, and there are only a few studies on the treatment and imaging of other diseases. Therefore, the following contents are aimed at drug-loading NBs. The most basic method for preparing NBs is the centrifugal separation of NBs from bubble solutions. Duan et al. [100] generated 200–700 nm NBs from a bubble solution through centrifugation. It not only can enter MBs that restrict access to tissues and capillaries but also facilitates local drug accumulation and disease therapy improvement. However, this form of NBs contains less gas, and thus ultrasonic reactivity is considerably decreased. The low gas loading of NBs can be solved by increasing the number of NBs in targeted sites, such as submicron bubbles or NBs with magnetic responses concentrate NBs in situ. External magnetic field intervention dramatically increases the concentration of local NBs, and this increase results in the desired cavitation effect induced by ultrasound, successfully opening the BBB and increasing the accuracy brain region targeting [101]. Chen et al. [122] embedded superparamagnetic iron oxide (SPIO) nanoparticles into the silicon shells of NBs for MRI/US dual-mode contrast agent imaging. A large number of therapeutic NBs were delivered to a specific brain region through an external magnetic field, and then ultrasonic irradiation was used to specifically target and open the BBB for medicine administration and brain tumor treatment. However, larger size of MBs and FUS beam can cause the opening of blood brain region through nonlinear oscillation without inertial cavitation, while in less size of NBs often produce inertial cavitation and affect the opening of BBB [25]. Magnetic nanomedicines preparation is cumbersome, NBs stability is poor, and precise specific equipment is required.

#### Ultrasound + PFCNDs + nanomedicines

**Fig.4:**
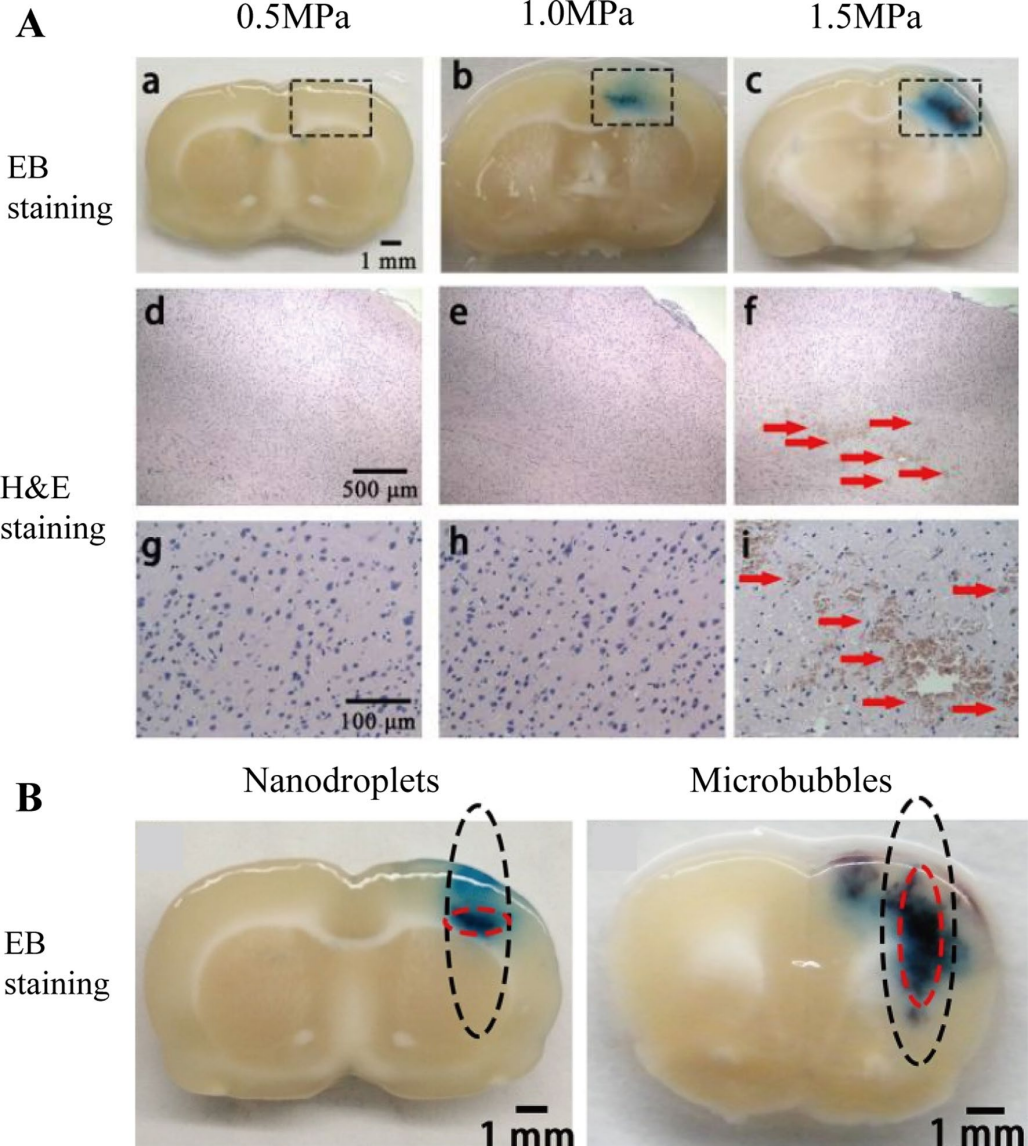
**A** FUS combined with PFCNDs induced BBB opening with acoustic pressure of 0.5 MPa, 1.0 MPa and 1.5 MPa. **a**–**c** EB staining of brain; **d**–**g** enlarged (× 40) corresponding black rectangle region in H&E stain; **g**–**i** enlarged (× 200) corresponding black rectangle region in H&E stain. **B** Distribution pattern of EB extravasation when nanodroplets or MBs was used as mediating agent combined with FUS [45]

Recently phase-change nanodroplets as PFCNDs have become the next-generation NBs, in which a perfluorocarbon-based phase change agent remains liquid at room temperature and vaporizes when ultrasound is applied. Correas and Quay first presented it in 1996 as a contrast agent, PFCNDs offer an attractive prospect for ultrasound diagnosis and drug delivery because of their unique phase transition capabilities, superior biocompatibility, and excellent tissue penetration [32, 102]. PFCNDs, similar to NBs, can also divided into two parts: drug-free or drug-loading PFCNDs. We hardly find drug-free PFCNDs for the brain tumors therapy, so the examples we discuss below are drug-loading PFCNDs. In PFCNDs, drugs can be combined or dissolved into the surfaces of lipid shells or deposited between shells and cores, and drugs dispensed in perfluorocarbon are sometimes encapsulated by nanodroplets. Eleanor Stride et al. [103] synthesized nanodroplets containing magnetic nanoparticles and a protein polymer shell. The nanodroplets remained active at body temperature for over 10 days and responded well to ultrasonic stimulation. Similarly, Liu et al. [130] prepared cationic nanodroplets loaded with PFP and nucleic acid. At low temperatures (25 °C and 37 °C), the nanodroplets were liquid. At 44 °C, MBs with a diameter of around 10 μm formed. Under ultrasonic irradiation, the nanodroplets opened the membrane barrier of HepG2 cells and increased gene transfection efficacy 14 times. Ultrasound-induced cationic phase-change nanodroplets can be considered excellent therapeutic agents for targeted gene delivery. As shown in the preceding cases, phase-change nanodroplets not only have a long in vivo circulation time (several hours in the human body) but also may be transformed into MBs in a particular position through ultrasound stimulation, thereby preventing MB blockage during vascular transportation. Furthermore, nanodroplets have a larger inertial cavitation threshold than that of MBs with a lower steady-state cavitation threshold, which is crucial for the effective and safe delivery of nanomedicines through the BBB [112]. Konofagou et al. [44] created MBs with a lipid shell coated with perfluorobutane and compressed them into nanodroplets to investigate the feasibility of these PFCNDs for brain-targeted drug delivery. The results indicated that when the ultrasonic sound pressure was 0.45 and 0.60 MPa, the delivery of model drugs to the brain has 60% and 100% efficiency, respectively. Compared with MBs, model drug delivery to the hippocampus via nanodroplets is more uniform and does not result in inertial cavitation. At normal atmospheric pressure, Cheng et al. [45] developed a type of nanodroplets composed of a PFP core and a polyethylene glycol-polylactic acid-glycolic acid shell. When the ultrasonic sound pressure was 1.0 MPa, the area of ethidium bromide (EB) overflow induced by the nanodroplets opening the BBB was strictly confined to the central small area and did not cause tissue damage, whereas the MBs caused a broad area of EB overflow, as shown in Fig. 4. These findings suggest that nanodroplets are viable alternatives to MBs and are promising tools for precisely managing BBB opening and drug delivery.

As previously described, ultrasound combined with MBs/NBs/PFCNDs brain delivery opens the BBB safely and efficiently. Given that the majority of carrier materials exhibits biocompatibility, suitable nanomaterial carriers must be further investigated because of the unique environment of the brain. Although the intervention of PFCNDs provides numerous advantages for brain drug delivery, it has drawbacks, including poor drug loading and single manufacturing procedure because of small particle size and the nonlipophilicity and hydrophobicity of fluorocarbons. The thermal and mechanical effects of cavitation may influence the therapeutic effect of nanomedicines. Moreover, PFCNDs are difficult to modify, and thus their brain tumor-targeting ability and performance and novel ultrasonic response materials should be investigated. Simultaneously, the metabolic pathways of these nanomaterials and drugs should be explored in vivo for the construction of brain drug delivery systems [104]. The safety of FUS warrants further study. Safe ultrasonic wavelengths and intensities should be determined. Finally, ultrasound assistance is a requirement for UABD nanomedicines application. Specialists on ultrasound equipment for the brain are rare, and this situation is undoubtedly a bottleneck hindering the utilization of the outstanding therapeutic effects of UABD nanomedicines. As a result, the creation of a specialized ultrasonic equipment for the treatment of brain diseases is an urgent problem of medical device developers.

In summary, UABD nanomedicines is a very effective brain drug delivery system for the treatment of brain diseases with great development potential. Although there have been some research reports on this area, the research on UABD nanomedicines is still in the primary stage in theory and technology due to the complex brain environment as well as the basis and technology of different disciplines such as ultrasound, medicine, material science and pharmacy. Many problems such as ultrasonic cavitation effect of UABD nanomedicines, BBB opening mechanism, cavitation materials and carrier design theory have not been solved, especially the properties of UABD nanomedicines after entering the brain have hardly been studied. The current UABD nanomedicines research is far from meeting the needs of clinical brain tumor therapy.

### Ultrasound response of the UABD nanomedicines

**Fig. 5:**
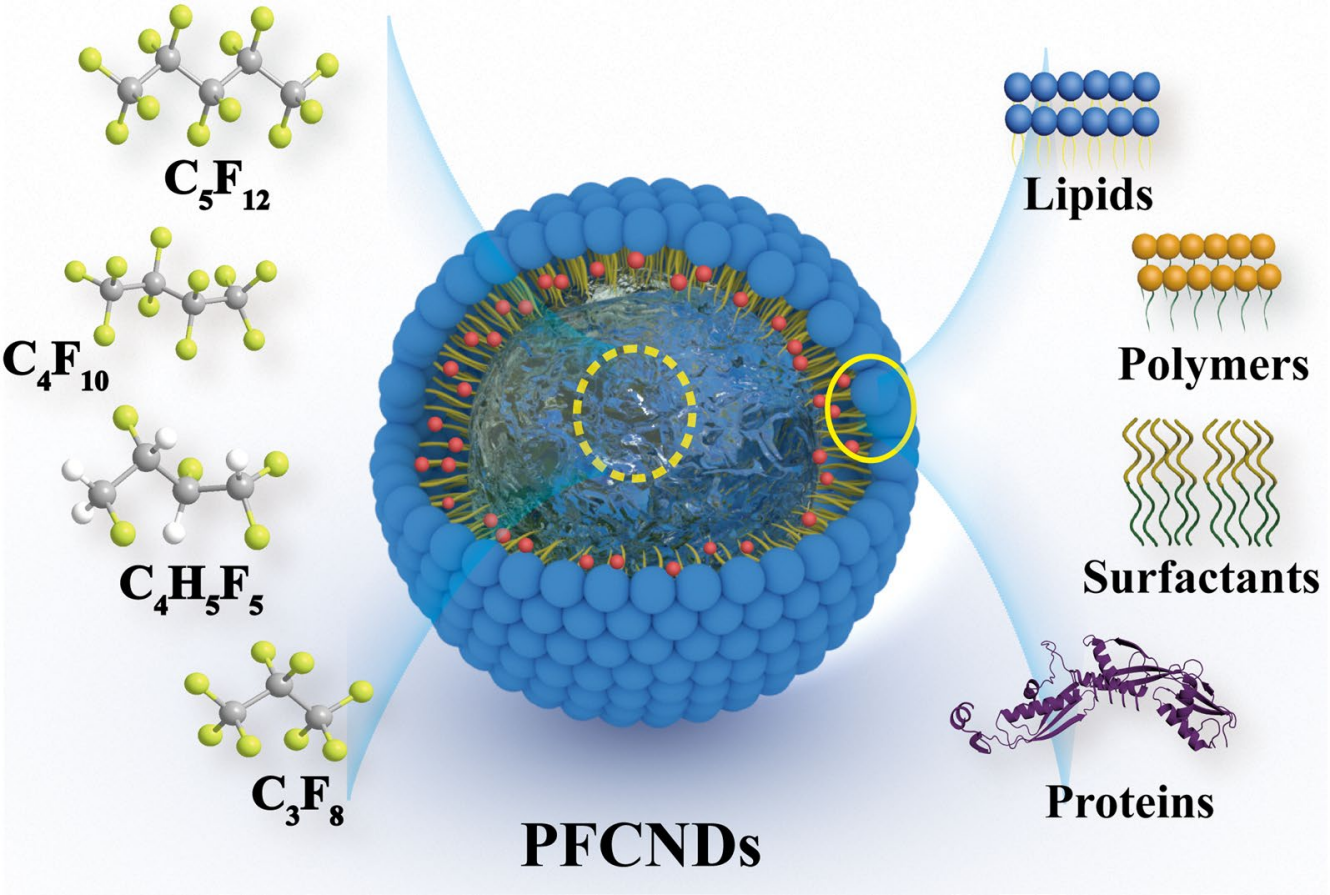
Structure and composition of PFCNDs

Apart from the commonly used carriers for nanomedicines, UABD nanomedicines contain unique cavitation materials that produce a specialized cavitation effect via the UABD technology. These unique cavitation materials include perfluorocarbon nanodroplets (PFCNDs), nanobubbles (NBs), and microbubbles (MBs). As shown in Fig. 5, PFCNDs or NBs are stable core–shell nanoparticles composed of perfluorocarbons (PFCs), such as perfluorohexane and perfluoropentane (PFP), and constitute the core, whereas lipids, polymers, proteins, and other substances, constitute the shell [105]. The physical properties of PFCs are determined by the number of carbon atoms (NCA). An NCA of < 5 indicates a gaseous state, and an NCA of ≥ 5 indicates a liquid state under normal conditions. External stimuli (such as temperature, pressure, or ultrasound) might induce a phase change in a PFC. Ultrasound is considered the most effective factor in promoting liquid-to-gas change in liquid PFCs [106]. This one-of-a-kind phase transition feature has been assumed to provide the prerequisite and foundation for cavitation [107].

At present, most PFCNDs are manufactured using a two-step emulsion process [108, 109]. PFCs are treated through emulsification for standby, and the emulsified PFCs are mixed with shell materials through a second emulsification process, and finally the excess carrier material is removed for the production of PFCNDs. Notably, drug-loaded PFCNDs can be prepared by dissolving drugs during the manufacturing process. For instance, Zhang et al. [110] first emulsified PFP and doxorubicin (DOX) and then mixed lipid membranes with the emulsion. DOX-loaded PFCNDs were collected through centrifugation. Interestingly, the positions of the drug-loaded PFCNDs varied depending on the shell material. Surfactant-encapsulated PFCNDs are typically placed between the shell material and PFCs, causing the drug to release instantly when the PFCND ruptures. Polymer-encapsulated PFCNDs are usually inside polymers and are thus slowly released even if the PFCs burst instantaneously; Additionally, the drugs can covalently attach to the surfaces of PFCNDs, and this feature enables drugs to stimulate a response. As a result, alternative drug-loaded systems might be selected according to requirements and maximize the therapeutic effects of drugs [111]. Moreover, small PFCNDs with long circulation facilitate drugs delivery through the BBB. The BBB can be opened, and cavitation nuclei form in tumor tissues via microflow passive or active extravasation under the action of ultrasound. This process facilitates the permeation of drugs into tumors. Concurrently, the phase shift of the PFCNDs generates MBs and can significantly increase the ultrasonic imaging effect, which is essential for disease detection and accurate therapy [112]. Generally, NBs/PFCNDs both form drug-loading nanoparticles when ultrasound combined with nanomedicines to treat brain tumor, so they are delivered into the body together with the drugs and reach the location of the BBB at the same time. In the administration process involving MBs, there are two cases: one is that MBs is administered intravenously together with the nanomedicines [7]; the other is that MBs is injected first and then administered within a certain period of time. In the former case, MBs and nanomedicines are usually injected at the beginning of the ultrasound; or ultrasound is performed 10–15 s after the injection of the MBs and nanomedicines [113]. In the latter case, ultrasound treatment was performed immediately after MBs injection, and then the BBB was opened, followed by the nanomedicines injection. According to our experience, it is better to conduct ultrasound treatment for 1 min immediately after the injection of MBs, followed by the injection of nanomaterials [114].

Another unique ingredient to UABD is the MBs carriers. Nanomedicines composed of common nanocarriers combined with MBs have the same cavitation capability as UABD nanomedicines. The majority of these common nanocarriers (liposomes and organic polymers) is biocompatible, minimizing the harmful or unpleasant effects of drugs during delivery [115]. MBs are micron-sized bubbles composed of core gases and shell materials, their shell ingredients are mainly non-ionic surfactants, albumins, lipids, polymers, and other materials, and filled gases include SF_6_, C_3_F_8_, C_4_F_10_, and other gases. The procedure for preparing MBs involves the dispersion of a gas to a micron-scale size and its wrap in a shell material. The product is a gas-carrying MBs structure. Sonication, high shear emulsification, membrane emulsification, inkjet printing, microfluidic processes, nanoparticle coalescence, and coaxial electrohydrodynamic atomization are some of the preparation technologies available [116]. Technical approaches for manufacturing MBs have been developed, and several commercial MBs (such as Sonovue) are frequently used in treating brain diseases. For instance, Liu et al. used Sonovue in combination with focused ultrasound to effectively open the BBB and cure central nervous system leukemia [117]. As shown in Table 3, we have summarized various parameters and the therapeutic efficacy of UABD nanomedicines. Most carriers used in brain tumor therapy are biodegradable and biocompatible with low toxicity, and the size distribution of carrier particles is relatively wide (ranges from tens of nanometers to 500 nm) [118, 119]. However, despite having a much smaller particle size than that of MBs, PFCNDs tend to clog tiny vessels because of phase change. Almost all drugs are released swiftly when PFCNDs reach a phase transition condition. Given that some drugs must be released slowly and constantly during clinical treatment, future studies on UABD nanomedicines should focus on the magnitude and continuous release of PFCNDs. For example, Meng et al. [131] synthesized doxorubicin-loaded PFCNDs, and the nanodroplets have a pH-sensitive chitosan-derived shell, which can respond to ultrasound and control the release of doxorubicin. Table 3 shows that UABD nanomedicines are mostly focused on the treatment of gliomas and relatively few researchers have studied tumor recurrence. Therefore, the use of UABD nanomedicines for other brain tumors, particularly large, medium, and advanced tumors, and other diseases should be systematically explored. In terms of therapeutic benefits in Table 3, even though the inhibition rate of UABD nanomedicines on glioma is more than 90% and the survival time can be prolonged by more than 20%, it is still far from completely cured, not to mention clinical application.

**Table 3:** The specific characteristics and therapy efficacy of UABD nanomedicines

Nanomedicines	Cavitation materials	Drugs	Particle size (nm)	tumors	Therapeutic effect	Refs
Liposomal Doxorubicin	MBs	Doxorubicin	∼100	Glioma	Survival time increased by 24%	[120]
PEG-AuNPs	–	Gold nanoparticle	50	–	–	[121]
MNBs	NBs	–	500	–	–	[122]
TMZ−NB@PLN−AAp	NBs	Temozolomide	400.3 ± 4.7	Glioblastoma	TIR: ~ 90%	[123]
DOX-CLs	Lipidosome (MBs)	Doxorubicin	≈180	Glioma	Survival extended to 81.2 days	[124]
PTX-LIPO	Lipidosome (MBs)	Paclitaxel	98.3	Glioma	Survival time increased by 20.8%	[125]
Albumin-Gd-DTPA	MBs	Gd	–	–	MR image enhancement; detect cerebral hemorrhage	[126]
Lipidosome-doxorubicin	Lipidosome (MBs)	Doxorubicin	≈100	Glioma	–	[127]
LPHNs_pCas9/MGMT_-cRGD	MBs	CRISPR/Cas9	179.6 ± 44.82	Glioma	Inhibit tumor growth and prolong the survival time of tumor-bearing mice	[128]
OFP and DFB droplets	PFCNDs	Proteins	171 /183	–	Successful delivery of 40 kDa dextran	[129]
Cationic nanodroplets	PFCNDs	Gene	300–400	–	Gene transfection efficiency was enhanced 14-fold on HepG2 cells	[130]
Acoustically-activated nanodroplets	PFCNDs	Dextran	200–300	–	Up to 33% of the animals showed a fluorescence enhancement	[44]

Thus, UABD nanomedicines design, drug delivery methods, and therapeutic approaches should be further improved for the treatment of intractable diseases. Fluorescence and magnetic resonance imaging (MRI) are primarily used in the treatment and diagnosis of glioma [132, 133]. For example, Yan et al. [134] used immunofluorescence staining technology to assess tumor cells and discovered that the group containing UABD nanomedicines had a higher proportion of apoptotic cells. These imaging or contrast agents may harm the human body during diagnosis. Additionally, MRI technology has drawbacks, including static imaging, and a lengthy scanning duration. Therefore, it should be explored for the incorporation of imaging capabilities into UAND nanomedicines and preparation of UABD nanomedicines with integrated diagnostic and therapeutic functions. This type of UAND nanomedicines will be absolutely essential for minimizing body damage during imaging and further boosting therapeutic efficacy.

### Therapeutic drugs of the UABD nanomedicines

The currently used therapeutic drugs of the UABD nanomedicines have been proved to be effective against brain diseases, including doxorubicin, gold nanoparticle, temozolomide, gene, etc. (as shown in Table 3). We can see that most therapeutic drugs are small molecule chemotherapeutic drugs, and only a small amount of biological macromolecular drugs such as genes, antibodies and proteins used for UABD nanomedicines [135]. In the future, we can foresee that it is very potential to deliver biomacromolecule drugs in this way and apply them to the treatment of brain tumors. Recently, Pan et al. used the cavitation of FUS combined with MBs to open the BBB and transfer the herpes simplex virus thymidine kinase gene to glioma. Thus, the anti-glioma effect of ganciclovir on tumor bearing rats was enhanced, and the survival time was longer [13]. In addition, antibodies or DNA fragments have been applied to the study of brain tumors on the base of UABD technology [136, 137]. However, due to the technical difficulties and the complexity of the brain environment, it is unclear which therapeutic drugs of the UABD nanomedicines are more suitable for brain tumor therapy, they need further research and verification.

## UABD nanomedicines for brain tumor therapy

Since the FDA approved FUS for the treatment of primary tumors in 2016, researchers have started exploring ways of using it in brain tumor treatment. In recent years, the UABD technology delivers drugs to the brain and diminishes tumor recurrence caused by invasive tumor cells and significantly increases the therapeutic effect of tumors [138]. However, the water solubility of the drugs is shown to limit the combination of UABD technology and free drugs, resulting in systemic side effects and decreased effective drug utilization. Owing to advancements in nanotechnology, UABD nanomedicines can efficiently address these inadequacies and have the potential use in brain tumor therapy.

Glioma is one of the most common primary intracranial malignant brain tumors with high recurrence and mortality. Glioblastoma (GBM) accounts for more than half of gliomas, which possess extremely invasive and extremely poor clinical prognosis. Although patients receive comprehensive treatment regimens, including chemotherapy, radiation, and surgery, the average survival time is 12–16 months, and more than 70–80% of patients die within 2 years [139]. Chemotherapy is considered an effective treatment for glioma, but the existence of the BBB prevents drug diffusion or delivery to the brain. Nanomedicines can improve efficiency in drug delivery through the BBB to the targeted brain region to a certain extent, but no overall improvement in therapeutic effect has been observed. The BBB can be opened with UABD technology, the delivery efficiency of various nanomedicines can be increased, and effective glioma therapy can be performed by using UABD nanomedicines [136].

### Ultrasound combined with MBs to deliver nanomedicines for the treatment of glioma

As UABD nanomedicines, MBs assisted by ultrasound successfully deliver nanomedicines to the brain for glioma therapy, which primarily relies on the ultrasound cavitation effect generated by the MBs to open the BBB and successfully deliver nanomedicines to the brain tumor. Subcutaneous and orthotopic tumorigenesis in naked mice are often used in the in vivo models of glioma. This basic procedure involves cultivating human or murine glioma cell lines and then injecting them into the brains or subcutaneous cultures of matched mouse strains until they develop into glioma. Subcutaneous tumors are routinely used in the early stages of research given that they are easy to observe and measure tumor size. Owing to the continuous development and progress in UABD technology, brain tumors are required to reflect real growing environment of tumors and drug metabolism. The paradigm of glioma has steadily transformed from subcutaneous tumor to orthotopic tumor in the treatment of glioma with UABD nanomedicines. The following examples all use ultrasound combined with MBs to deliver nanomedicines to in situ glioma models in the brain.

**Fig. 6:**
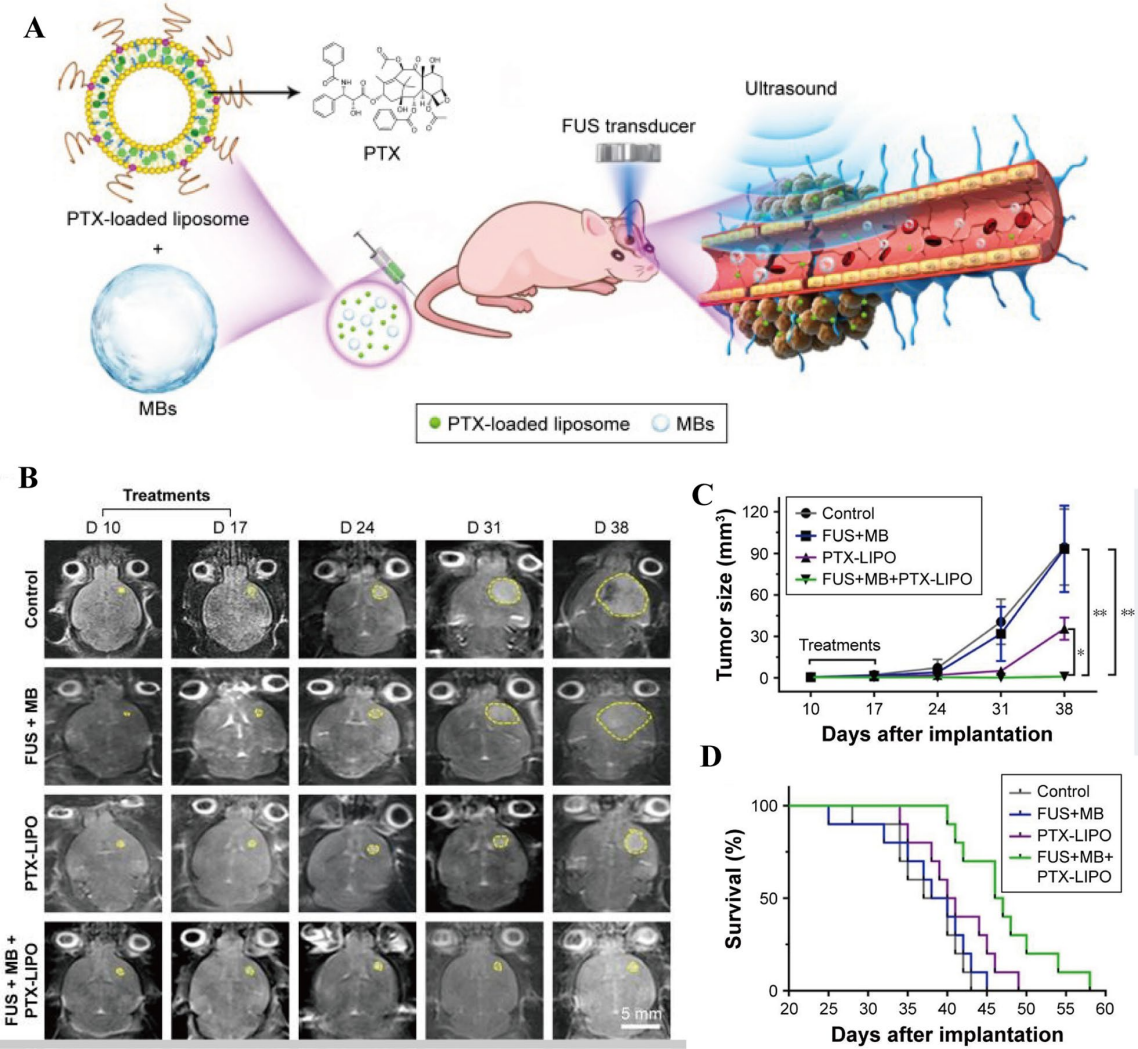
**A** Schematic illustration of brain tumor delivery PTX-LIPO by USCM. **B** Representative T2-weighted MRI horizontal images of intracranial U87 MG glioblastoma progression (yellow outline) before and after different treatments. **C** Tumor volumes from day 10 to day 38 after implantation of nude mice with different treatments. **D** The Kaplan–Meier survival curves of nude mice in each treatment group [125]

For example, Zheng et al. [140] prepared organic–inorganic hybrid hollow mesoporous organosilicon nanoparticles (HMONs), where disulfide linkages bind ultra-small Cu_2_-xSe nanoparticles to the surface (HMONs-ss-Cu_2_-xSe, abbreviated as HCu). The DOX was then encapsulated in the hollow structure of HCu for the construction of DOX-HCu nanomedicines. The DOX-HCu and MBs were injected via the tail vein into mice with GBM. The BBB is opened under the cavitation effect produced by ultrasound combined with MBs, the DOX-HCu was accurately delivered to the GBM and penetrated tumor tissues. The results showed that the tumor growth inhibition rate (91.1%) was significantly higher than that in the free DOX group (35.4%), the free DOX combined with ultrasound group (69.2%), or DOX-HCu group (52.4%), indicating that the nanomedicines is extremely effective in glioma treatment. Additionally, the studies illustrated that the hollow organic mesoporous silicon nanoparticles have stable structures, excellent biocompatibility, and biodegradability. However, the metabolic mechanism of nanoparticles in vivo has not been thoroughly examined, and thus whether residual nanoparticles that induce brain tissue damage are present is unclear. Liposomes as carriers are commercially available, and the metabolic mechanism is quite clear. Chen et al. [125] developed paclitaxel-liposome nanomedicines (PTX-LIPO) by encapsulating paclitaxel in the phospholipid bilayers of liposomes, as shown in Fig. 6. With the aid of UABD technology, the growth of intracranial glioblastoma in nude mice in different treatment groups can be seen in Fig. 6B and C, the tumor inhibition rate of the FUS + MB + PTX-LIPO group was as high as 99%, and the median survival time of mice in this group increased by 20.8%. This result shows that substituting a carrier material with liposomes increases the capacity of brain tumors and decreases damage to normal tissues. However, histological slices of the tumor revealed that the maximum level of tumor cell apoptosis in the FUS + MBs + PTX-LIPO group was only 77.5%, implying a high risk of tumor recurrence, which was not explored further in this study. To further improve the therapeutic effect of brain tumors and avoid tumor recurrence, targeted function is usually introduced. Chen et al. [79] integrated magnetic guidance into UABD nanomedicines. They immobilized 1,3-bis (2-chloroethyl)-1-nitrosourea (BCNU) on magnetic nanoparticles (MNPs) to prepare BCNU-MNPs. Guided by an external magnetic field, these magnetic targeting nanomedicines can dramatically boost the efficiency of antitumor drug delivery to brain. Inductively Coupled Plasma-Optical Emission Spectroscopy (ICP-OES) analysis revealed that the accumulation of BCNU-MNPs combined with magnetic field or ultrasound was about 21 times that of BCNU-MNPs combined with ultrasound in normal rat brains. Moreover, tumor inhibition in mice treated with high-dose BCNU (5 mg/kg) was as high as 97% after one week. However, the survival time of high-dose mice is not as long as that of medium dose (1 mg/kg), which may be due to the high toxicity of high-dose drugs to organs. This shows the therapeutic effect needs to be comprehensively considered even though the introduction of magnetic targeting technology can significantly increase the accumulation of drugs in the brain. Wan et al. [114] designed an all-in-one nanomedicine (AMPTL) composed of paclitaxel (PTX), 3-methyladenine (3-MA), and angiopep-2 peptide by cascade-amplifying synergistic therapeutic strategy. Cytometry results showed that the autophagy inhibitor 3-MA caused more tumor cells to remain in the G2/M phase. Under UABD technology, the efficiency of PTX in permeating the BBB reached 30.58%, which was approximately 22 times that of the non-irradiated controls group (1.39%). Finally, H&E staining of brain tissues revealed that the AMPTL + PLUS + MBs group exhibited a strong tumor suppressive effect, with a median survival period of 50 days, versus the 28 days in the untreated group. Hence, combining alternative therapies with chemotherapy appears to be a promising treatment strategy for glioma.

**Fig. 7:**
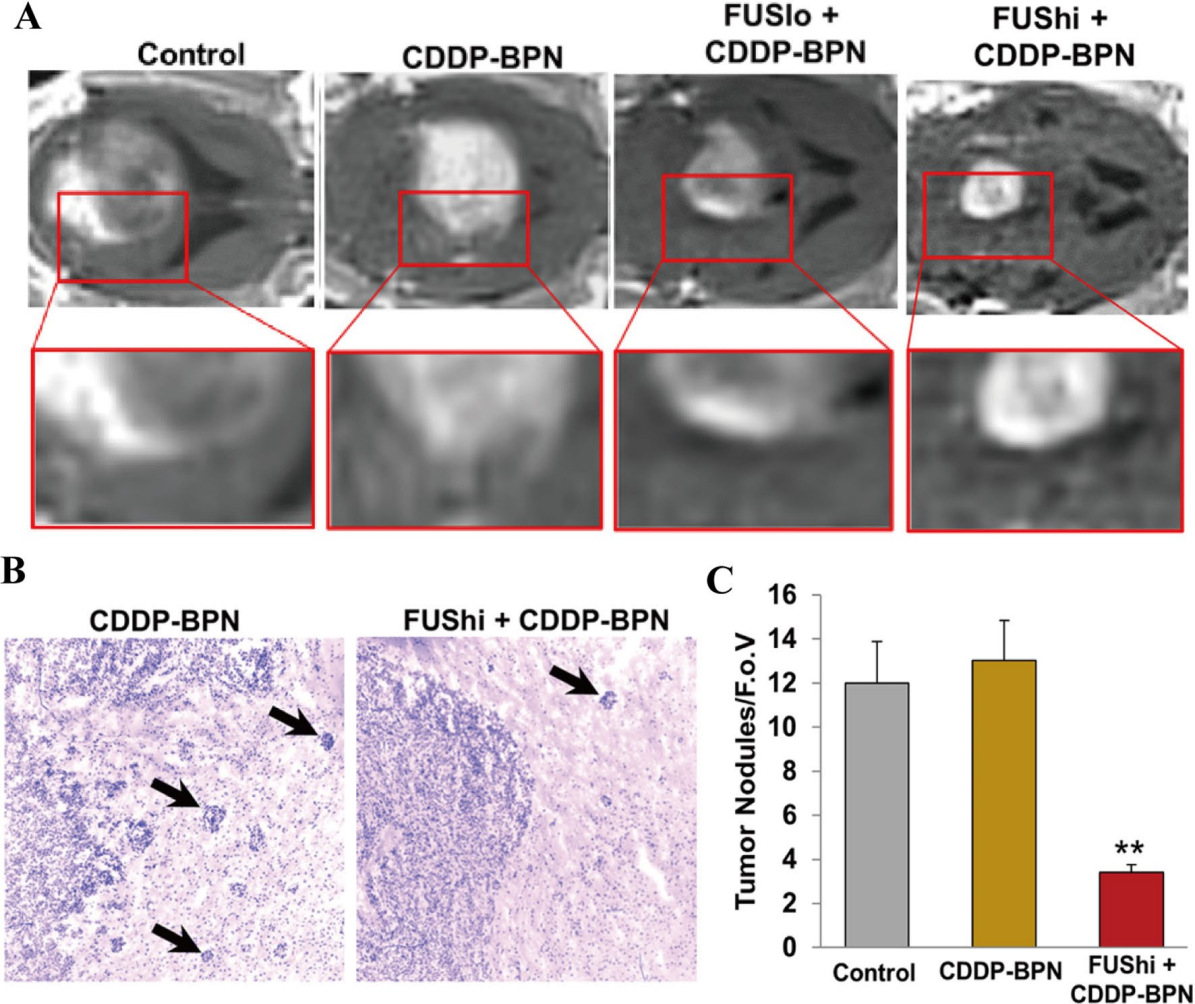
**A** Animals treated with high intensity FUS in combination with BPN nanomedicines had smaller tumors with more defined borders. **B** the tumor border in the FUShi + CDDP-BPN image is well-defined compared to the CDDP-BPN image. Infiltrating tumor nodules are denoted with black arrows. **C** Bar graph of infiltrating tumor nodules per field of view [51]

Some researchers have conducted pertinent study on invasive glioma in situ. Price et al. [51] developed cisplatin-loaded polymer nanoparticles (CDDP-BPN) that was stable in the blood for up to 24 h. The efficiency of MRgFUS-mediated CDDP-BPN in crossing the blood tumor barrier was six and 28 times that of the control group in the 9 L glioma and invasive F98 glioma models, respectively. When FUS was applied at a pressure of 0.8 MPa, the edge of the F98 tumor treated with the CDDP-BPN was more visible, and the number of invasive tumor nodules outside the tumor edge was reduced 3.5 times, as shown in Fig. 7. These findings showed that using the MRgFUS to transport these nanomedicines from the systemic circulation to F98 gliomas significantly reduces tumor invasion and growth while improving the survival rates of animals. We can conclude that ultrasound combined with MBs administration is extremely effective and safe for the treatment of intracranial glioma and is likely to be employed in clinical treatments in the future. However, MBs’ stability in vivo is not optimal, and they tend to microvascular obstruction because of their large volumes. To address this issue, application research on NBs and PFCNDs has been undertaken.

### Ultrasound combined with NBs to deliver nanomedicines for the treatment of brain tumor

**Fig. 8:**
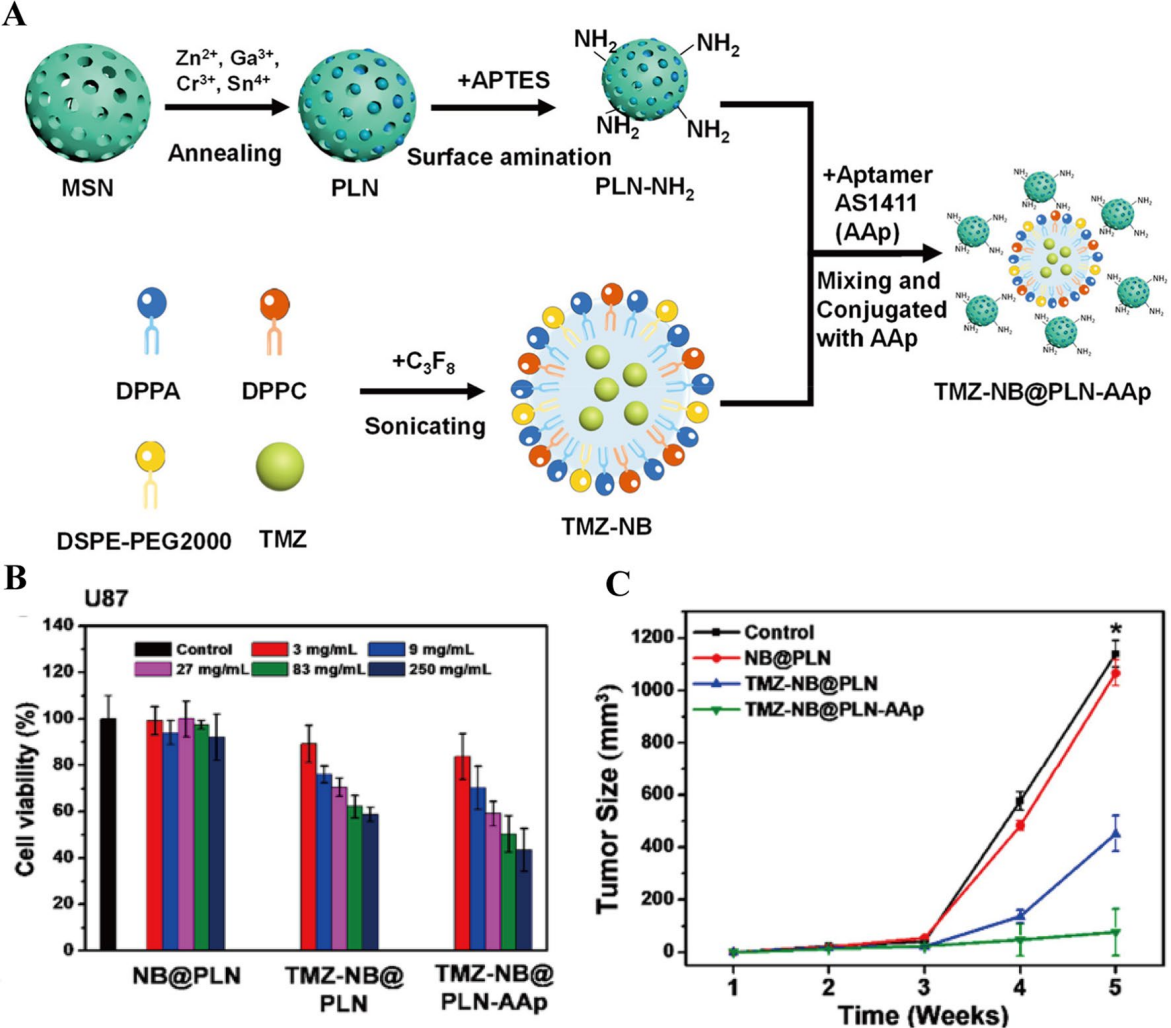
**A** The preparation and surface functionalization of TMZ-NB@PLN-AAp. **B** U87 cell viability after incubation with different concentrations of TMZ-NB@PLN and TMZ-NB@PLN-AAp. **C** Quantification of the tumor sizes after different treatments over time [123]

NBs/PFCNDs have gradually supplanted MBs in UABD nanomedicines and play a critical supporting role in the treatment of brain tumors. On the one hand, the size advantage of NBs not only eliminates the risk of blood vessel blockage but also significantly improves the efficiency of medicines in traversing various bodily barriers. On the other hand, NBs have more flexible modification ability than MBs, offering a wide range of possibilities for NBs functional development. On the basis of these features, researchers used ultrasound combined with that of NB_S_ to deliver nanomedicines for glioma treatment. Owing to the immature state of the research, the subcutaneous glioma model was selected for the study during the treatment process. As shown in Fig. 8A, Hsiao et al. [123] made a series of studies in this area. They embedded TMZ in NBs and allowed them to interact with nanoparticles (PLNs) through electrostatic interaction to prepare TMZ-NB@PLN-AAp with a size of 400.3 ± 4.7 nm. The results of cell experiment revealed that the mortality of glioma cells treated with 83 and 250 mg/ml TMZ-NB@PLN-AAp reached 50% and 60%, respectively (Fig. 8B). Besides, Fig. 8C shows that the growth of subcutaneous gliomas was almost stagnant after 5 weeks of therapy with TMZ-NB@PLN-AAp, and the tumor size was reduced by about 90% relative to that in the control group. Changes in the growth of brain tumors in mice over a 3 week-period were tracked using a brain in vivo imaging system. The tumors of mice treated with TMZ-NB@PLN-AAp drastically decreased. This beneficial therapeutic effect was attributed not only to the cavitation effect produced by ultrasound combined with NBs that broke the BBB but also to the passive accumulation of NBs at the tumor site by the targeting of AAP. In another experiment, they loaded FePt nanoparticles and DOX into the hydrophobic cores of NBs to create a Dox-FePt@NB drug delivery system [141]. The surfaces of THE NBs were then modified with a targeting ligand-transferrin complex to generate Dox-FePt@NB-Tf with a size of 200–300 nm. These nanomedicines actively targeted and accumulated in the brain under the influence of a magnetic field. The cavitation effect caused the BBB to momentarily open, facilitating the entry of drugs into the brain and treatment of brain tumors. The sizes of the glioma mice treated with DOX-FePt@NB-Tf group decreased by about 70% after 5 weeks, and the survival rates of the mice were 3.5 times those of the control group after 10 weeks of observation. The main reason was that the tumor-specific targeting ligand-Tf complex greatly increased the targeting effect of magnetic nanoparticles. When combined with the induction of an external magnetic field, Dox-FePt@NB-Tf exhibited significant antitumor effects. The use of NBs increased the drugs content at the tumor site, but the tumor inhibition rate did not improve compared with that of MBs. The possible reason was that the cavitation effect of the NBs exposed to ultrasound was limited, and hence the actual content of drugs that passed through the BBB was relatively low. Moreover, the subcutaneous glioma model was used in all cases, which did not accurately reflect the real environment of in situ glioma formation as it lacks the environment of the BBB and immune privilege sites. Furthermore, the tumor recurrence occurred in glioma mice receiving ultrasound combined with NBs therapy, which is mainly manifested in a significant tumor inhibition effect within 1 week, but the tumor volume increased over time.

PFCNDs have not been employed for the treatment of brain tumors, although they have been used as drug carriers and contrast agents for ultrasound imaging in the treatment of other tumors. IR780-ND_S_ nanomedicines were created by Li et al. [142] Lipophilic IR780 was incorporated into lipid bilayer and PFP was encapsulated in the core. PFCNDs are transformed into MBs with ultrasound irradiation, which can produce vascular rupture and tissue erosion, allowing more IR780-ND_S_ to leave the systemic circulation into the tumor stroma and penetrate into tissues further away from the blood vessels. In the control, US-only and IR780-NDs-only groups, the tumors grew rapidly without any therapeutic effect. Tumor growth was significantly inhibited in the US + IR780-NDs group, and the tumor volume after 16 days of treatment was roughly one-third that of the control group. Additionally, Zhang et al. [110] developed PFCNDs to act as nanocarriers for DOX and indocyanine green (ICG) to construct PFP/ICG/DOX@LIP nanodroplets. In this study, PFCNDs transformed into highly echogenic bubbles was demonstrated to be effective ultrasonic imaging probes for the controlled delivery and release of DOX through light-controlled US imaging.

As demonstrated by the preceding two studies, PFCNDs as nanocarriers offer great advantages and development potential. They play a critical role in drug delivery for tumor therapy and facilitate the use of ultrasonic imaging. However, this field of research is still in its infancy and requires continued exploration, especially in the treatment of brain tumors. Under the radiation of ultrasound, PFCNDs rapidly expanded into MBs and open the BBB with cavitation, their carried drugs released meanwhile. The BBB pass efficiency of free drugs are significantly reduced than that of nanomedicines after MBs opening the BBB. This may be the reason why PFCNDs are difficult to treat brain tumors at present. Many challenges must be overcome in the application of PFCNDs to brain tumor therapy. Restrictions on the skull and develop a suitable method for the in situ monitoring of brain tumor progression. The functional modification of PFCNDs should be explored further to ensure that UABD nanomedicines keep their tumor targeting ability after opening the BBB and ensure the nanomedicines into the brain. After resolving these issues, we can predict that PFCNDs will demonstrate a satisfactory therapeutic effect for the treatment of brain tumors.

UABD nanomedicines have the advantages of high drug delivery efficiency, few side effects, enhanced targeting, and increased safety for brain tumor therapy. Ultrasound combined with the MBs delivery of nanomedicines for the treatment of glioma is a particularly successful technology that can significantly improve the efficiency of brain drug delivery and ensure the effective treatment of glioma in situ. Moreover, although ultrasound combined with NBs/PFCNDs delivery of nanomedicines can overcome the problems associated with the large sizes of MBs, the degree of BBB opening is relatively unsatisfactory, and orthotopic glioma have not been studied in this field. The possible reason is that the NBs/ PFCNDs have relatively low drug loading and BBB pass efficiency because of their sizes and are not conducive to various modifications. The effects of opening the BBB have not been thoroughly explored. If these problems were solved, NBs or PFCNDs would become an important therapeutic tool in the future.

## Challenges and prospect

Nowadays, ultrasound combined with MBs is the only way to open BBB effectively and safely. Combining this method with nanomedicines (UABD nanomedicines) has become a potential treatment strategy for brain tumors. Because ultrasound and some nanomedicines have been used in clinic, this strategy has immeasurable application prospects in the future. However, due to the knowledge and skills of various disciplines involved in this field, its research is still in the primary stage. In particularly, less studies on UABD nanomedicines have been reported, while ordinary nanomedicines are difficult to meet the requirements of brain drug delivery. Systematic research on the relationship between nanomedicines and ultrasound-assisted brain delivery, such as the interaction between nanomedicines and BBB, ultrasound and MBs cavitation, will provide a basis for the design and improvement of UABD nanomedicines. Therefore, according to these results, it is of great theoretical and practical significance to develop new UABD nanomedicines with high BBB passing rate and low side effects. Apart from the treatment of brain tumors, UABD nanomedicines play an important role in the therapy of other central nervous system diseases [129]. For example, Liu et al. [54] and Price et al. [143] used UABD technology to briefly open the BBB and successfully transfected the gene fragments carried by GDNFp into central nerve cells to promote neuronal growth and development, resulting in significant improvement in the behavior of mice with Huntington's disease and Parkinson's disease. Banks et al. [144] and Green et al. [48] loaded anesthetic drugs inside PFCNDs, which not only successfully delivered the drugs to the central nervous system lesion but also accomplished the purpose of accurate controlled release by adjusting ultrasonic parameters. Although ultrasound combined with PFCNDs has not been employed in the treatment of brain tumors, it has been used in the therapy of neurological diseases, as seen in the case above. As a result, ultrasound combined with PFCNDs will be a promising therapy option for brain diseases therapy. We can deduce from the preceding chapters that MBs, NBs, and PFCNDs can open the BBB in response to ultrasound irradiation, enabling the transport of nanomedicines and the treatment toward certain brain tumors. On the basis of a comprehensive analysis of UABD nanomedicines and their applications, the following issues and challenges with UABD nanomedicines for brain tumor therapy are clarified:

1. UABD nanomedicines lack theoretical support. Ultrasound combined with nanomedicines has been associated with many theories of differed disciplines and the carrier materials of ultrasound response have been systematically studied and summarized. However, UABD nanomedicines are merely built on the design experience of other nanomedicines domains. More appropriate and unique carrier materials for UABD nanomedicines is urgently required for brain tumor therapy. In addition, no comprehensive review of UABD nanomedicines and their mechanisms has been conducted. Therefore, appropriate UABD nanomedicines should be developed, and existing theories should be supplemented. Another critical point is that present research is primarily focused on how to penetrate the BBB, and few studies evaluated the effects of nanomedicines once they enter the brain. Multiple studies failed to address this issue at all and instead described the treatment effects in general. Understanding the effects of nanomedicines on the brain will be a critical component of future research.
2. MBs/NBs/PFCNDs have obvious limitations. As multiple studies have revealed, the assistance of MBs/NBs or PFCNDs is a necessary condition for ultrasound to safely open BBB [26]. While MBs and liposomes are the only two types of ultrasound-responsive materials that have undergone clinical trials possibly because of their good biocompatibility and low toxicity. As mentioned above, the application of MBs and NBs for the treatment brain tumors have numerous problems. Many brain studies have shifted their focus to PFCNDs to increase the accuracy and efficiency of the treatment process [145], the size of PFCNDs is much smaller than that of MBs and amounts of perfluorocarbons loaded is limited. Given that the actual concentration of the perfluorocarbons entering the BBB or brain is still relatively low, their BBB opening effect is inferior to that of MBs. Meanwhile, delivery of nanomedicines by PFCNDs have been studied in other tumors and even other brain diseases but have not made relevant progress in brain tumors. The most reasonable explanation is that the PFCNDs’ medicine loading capacity is insufficient to meet the needs of brain tumors’ therapy.
3. Specific ultrasound equipment for BBB opening is limited. Medical ultrasound equipment has been commonly implemented in disease diagnosis and therapy. No specific ultrasonic equipment that facilitates the opening of the BBB and the delivery of nanomedicines to the brain is available. Furthermore, the ultrasonic equipment and parameters setting used to treat brain diseases at this stage are different, and thus the degree of opening the BBB varies depending on them. Instrument research in this field necessitates the collaboration of researchers from multiple disciplines, such as ultrasound, medicine, brain science, materials science, pharmacy, and others, which lead to the complexity of instrument research. As a result, brain-specific ultrasound equipment should be developed and studied.
4. Treatment effect of glioma is unsatisfactory. Glioma is one of the most common brain tumors, as we can see from the preceding description, extensive research on the treatment of glioma with MBs combined with nanomedicines triggered by ultrasound has been studied, and the therapeutic effect is pretty apparent. However, the continuous optimization of ultrasound parameters and further research on recurrent tumors, large and medium-sized tumors, are essential. Although UABD technology can improve nanomedicines delivery efficiency and prolong the survival time of patients, it is still the primary stage of brain tumor research. Glioblastoma has a high recurrence rate and poor prognosis, so researchers should conduct more in-depth and comprehensive studies on the treatment of glioma at all stages.
5. Clinical research is in the preliminary stage. Clinical experiments have proved that it is feasible to open the BBB temporarily in glioblastoma (GBM) by FUS combined with systemic perfusion MBs. There is a new device NaviFus for this application, which integrates neuronavigation and FUS-MBs system. Wei et al. found that it can accurately and repeatedly guide ultrasonic energy to the target central nervous region during operation and minimize side effects [146]. In addition, Carpentier et al. [147] adopted an implantable ultrasound equipment system for patients with brain tumors to destroy the BBB with the help of MBs, and delivered the chemotherapeutic drug carboplatin into the brain to treat recurrent GBM. Unfortunately, for ethical reasons, the current study design does not allow the measurement of carboplatin concentration in the brain through additional post-treatment procedures. In the mouse model of glioblastoma, we observed an increase in the survival time model of glioblastoma treated with these chemotherapeutic drugs [148]. It can be seen that there were no serious adverse events when ultrasound combined with MBs delivered free drugs to treat brain tumors. However, there is no clinical study on the application of UABD nanomedicines in the treatment of brain tumors. On the one hand, it may be that the application of UABD technology in the complex environment of brain is not mature enough; On the other hand, the properties and types of nanomedicines more suitable for the brain need further research. In the future, it is believed that UABD technology will be the clinical trend in the treatment of brain tumors, and nanomedicines will also replace free drugs to further improve the treatment effect, although these results need to be confirmed in further research [102, 149].

Based on these points, we propose future work to focus on the following research directions:

1. Developed multifunctional and high-performance UABD nanomedicines. We believe that the primary goal of this research is to develop high-performance UABD nanomedicines. On the one hand, we should explore new nanomaterial and preparation methods to make the carrier size more suitable for the brain’s environmental requirements after systematic research on the influence of BBB and ultrasonic cavitation for UABD nanomedicines [150]. On the other hand, drugs can self-assemble into nanoparticles of infinite coordination polymers/metal–organic frameworks/ covalent-organic frameworks; and those type of nanomedicines have not yet been applied to the brain tumor therapy, which will be a significant research field of brain [151]. Moreover, the further development of multifunctional UABD nanomedicines carriers to achieve multiple functions, such as targeted transport and synergistic therapy, which can greatly improve the therapeutic effect of brain tumors, is expected to become a major subject of interest. Magnetic targeting, optical imaging, and other features can be integrated into nanomedicines to achieve the purpose of one objection with multi-functions. The construction of multifunctional nanocarriers usually demand the use of a variety of materials, which not only increases the complexity of the preparation process but also often brings potential safety hazards. Therefore, our ultimate goal is to construct more efficient and safer multifunctional nanocarriers. A novel type of UABD nanomedicines carrier with an “integrated diagnostic and therapeutic system” should be developed and manufactured, which can realize a real-time and accurate diagnosis of the disease and simultaneous treatment it. It can monitor the curative effect and adjust the administration plan at any time throughout treatment, which is conducive to achieving the best treatment effect and minimizing adverse effects.
2. Optimize the performance of MBs/NBs/PFCNDs. The stability of MBs, NBs, and PFCNDs should be maintained in vivo. Commercial MBs have a service life of about 5 min, and thus it does not meet the needs of disease therapy. Simultaneously, the boiling point of PFP in the PFCNDs core is 29 ℃, which turns into a gaseous phase when injected into the physiological state of human body; and the boiling point of perfluorohexane is higher, which requires higher ultrasonic energy to promote the liquid–gas phase transition. Thus, two strategies could improve the stability of MBs, NBs, and PFCNDs in vivo. On the one hand, they can be surface-modified to extend their service life. On the other hand, fluorocarbons currently used can be modified to make their phase-transition temperature match the treatment environment and respond to ultrasound more accurately. In addition, expanding the function of MBs, NBs, and PFCNDs can improve the therapeutic effect. For instance, the introduction of fluorescent molecules or magnetic nanoparticles onto MBs, NBs, and PFCNDs can achieve real-time monitoring and magnetic targeting. Improving the stability of MBs, NBs, and PFCNDs by various means of innovating the existing materials is a vital method. Ultrasound combined with PFCNDs has considerable application potential in brain drug delivery and brain disease therapy, but there is no relevant reference case for the treatment of brain tumors is currently available. Researchers should accelerate the pace to explore the relevant mechanism of ultrasound combined with PFCNDs in the treatment of brain tumors. Various problems should be resolved. Scholars from diverse disciplines will be attracted to establish the foundation for this field’s sustainable development.
3. Development of specific ultrasound equipment. The ultrasonic equipment in UABD nanomedicines should meet the requirements of accurate and stable output of sound intensity and frequency, convenient operation, safe modalities, painless approach, and obvious therapeutic results. Thus, the research and development of brain ultrasound equipment requires collaboration between researchers in ultrasound, medicine, brain science, and other fields. One of the reasons for the limitations of UABD nanomedicines application is lack of specialized equipment. The treatment of UABD nanomedicines for brain tumors is only limited to the treatment of gliomas therapy, and studies for the treatment of other brain tumors are few. As a result, a specific ultrasound equipment for UABD nanomedicines should be developed, laying a solid foundation for deepening the treatment of gliomas and other diseases, as well as future clinical transformation.
4. Study on the specific mechanism of the UABD technology opening BBB and reducing body injury. These available techniques and instruments are insufficient to enable comprehensive observation and research of the process by which the UABD technology opens the BBB. Thus, further study on the mechanism by which UABD technology opens the BBB is necessary, along with the continuous development in other technologies. How to precisely prevent damage the BBB during the process of UABD technology is a critical research direction and is also a key step in the translation of this technology into clinical applications. It is required to elucidate the metabolic pathways, distributions, and side effects of nanomedicines in the body to build the theoretical foundation for the development of brain drug delivery systems.
5. Construct a synergistic treatment strategy. At present, although there are various types of drug carriers, many MBs carriers cannot target any local tissue or specific lesion site after intravenous injection [152]. D'Arrigo et al. took UABD technology combined with "lipid-coated MBs (LCM) /nanoparticle-derived" lipid nanoemulsion with cell surface lipoprotein receptor SR-BI as the target to treat Alzheimer's disease, so that Alzheimer's patients better treatment outcomes were obtained [153]. Under the action of ultrasound, the cavitation effect produced by MBs opens the BBB, and then the therapeutic agent will target the cell surface SR-BI to achieve the purpose of enhancing the effect of local drug therapy on brain tissue in vivo. In addition, there are also studies using magnetic targeting to make drugs gather more at the tumor site and play a better therapeutic effect on brain tumors [135]. However, up to now, there are few studies using UABD technology combined with other targeting methods, especially in the research of treating brain tumors. Therefore, this research field is very worthy of research and full of prospects.

## Conclusion

UABD nanomedicines is a novel strategy for brain drug delivery, which can effectively and safely treat brain tumors with therapeutic drugs and has broad clinical translation prospects. MBs/NBs/PFCNDs or other specific materials are needed as the institutes of UANBD nanomedicines, but each of them has drawbacks, such as large particle size of MBs and single preparation method of PFCNDs. Although UABD nanomedicines have demonstrated promising therapeutic effects for brain tumors therapy, several issues of UABD nanomedicines remain unresolved. Researchers in different fields need to work together to overcome these problems and make considerable progress in this field.

## Data Availability

All data generated or analyzed during this study are included in this published article (and its supplementary information files).

## Abbreviations

BBB: Blood–brain barrier
UABD: Ultrasound-assisted brain delivery
HIFU: High-intensity focused ultrasound
USCM: Ultrasound combined with microbubble
MBs: Microbubbles
AD: Alzheimer's disease
FUS: Focused ultrasound
PFCNDs: Perfluorocarbon nanodroplets
NBs: Nanobubbles
PFCs: Perfluorocarbons
PFP: Perfluoropentane
NCA: The number of carbon atoms
DOX: Doxorubicin
TMZ: Temozolomide
GBM: Glioblastoma multiforme
MRI: Magnetic resonance imaging
VEGF-A: Vascular endothelial growth factor
BCNU: 1,3-Bis (2-chloroethyl)-1-nitrosourea
SPIO: Superparamagnetic iron oxide
EB: Ethidium bromide
HMONs: Hollow mesoporous organosilicon nanoparticles
PTX-LIPO: Paclitaxel-liposome nanomedicines
MNPs: Magnetic nanoparticles
3-MA: 3-Methyladenine
ICG: Indocyanine green
GDNFp: Glial cell line-derived neurotrophic factor plasmid
